# CAF hierarchy driven by pancreatic cancer cell p53-status creates a pro-metastatic and chemoresistant environment via perlecan

**DOI:** 10.1038/s41467-019-10968-6

**Published:** 2019-08-12

**Authors:** Claire Vennin, Pauline Mélénec, Romain Rouet, Max Nobis, Aurélie S. Cazet, Kendelle J. Murphy, David Herrmann, Daniel A. Reed, Morghan C. Lucas, Sean C. Warren, Zehra Elgundi, Mark Pinese, Gabriella Kalna, Daniel Roden, Monisha Samuel, Anaiis Zaratzian, Shane T. Grey, Andrew Da Silva, Wilfred Leung, Australian Pancreatic Genome Initiative (APGI), Amber L. Johns, Lorraine A. Chantrill, Angela Chou, Angela Steinmann, Mehreen Arshi, Tanya Dwarte, Danielle Froio, Brooke Pereira, Shona Ritchie, Cecilia R Chambers, Xanthe Metcalf, Nicola Waddell, John V. Pearson, Ann-Marie Patch, Katia Nones, Felicity Newell, Pamela Mukhopadhyay, Venkateswar Addala, Stephen Kazakoff, Oliver Holmes, Conrad Leonard, Scott Wood, Sean M. Grimmond, Oliver Hofmann, Angelika Christ, Tim Bruxner, Jaswinder S. Samra, Nick Pavlakis, Hilda A. High, Ray Asghari, Neil D. Merrett, Darren Pavey, Amitabha Das, Peter H. Cosman, Kasim Ismail, Chelsie O’Connnor, Alina Stoita, David Williams, Allan Spigellman, Vincent W. Lam, Duncan McLeod, Judy Kirk, James G. Kench, Peter Grimison, Caroline L. Cooper, Charbel Sandroussi, Annabel Goodwin, R. Scott Mead, Katherine Tucker, Lesley Andrews, Michael Texler, Cindy Forest, Krishna P. Epari, Mo Ballal, David R. Fletcher, Sanjay Mukhedkar, Nikolajs Zeps, Maria Beilin, Kynan Feeney, Nan Q. Nguyen, Andrew R. Ruszkiewicz, Chris Worthley, John Chen, Mark E. Brooke-Smith, Virginia Papangelis, Andrew D. Clouston, Andrew P. Barbour, Thomas J. O’Rourke, Jonathan W. Fawcett, Kellee Slater, Michael Hatzifotis, Peter Hodgkinson, Mehrdad Nikfarjam, James R. Eshleman, Ralph H. Hruban, Christopher L. Wolfgang, Rita T. Lawlor, Stefania Beghelli, Vincenzo Corbo, Maria Scardoni, Claudio Bassi, Andrew V. Biankin, Judith Dixon, Nigel B. Jamieson, David K. Chang, Suresh Mathivanan, Yingxiao Wang, Anthony W. Braithwaite, Daniel Christ, Ales Benda, Ashleigh Parkin, Phoebe A. Phillips, John M. Whitelock, Anthony J. Gill, Owen J. Sansom, David R. Croucher, Benjamin L. Parker, Marina Pajic, Jennifer P. Morton, Thomas R. Cox, Paul Timpson

**Affiliations:** 1 The Garvan Institute of Medical Research & The Kinghorn Cancer Centre, Sydney, NSW 2010 Australia; 2 St Vincent’s Clinical School, Faculty of Medicine, University of New South Wales Sydney, Sydney, NSW 2010 Australia; 3 Molecular Pathology department, the Netherlands Cancer Institute, Amsterdam, 1066CX the Netherlands; 4 Graduate school of Biomedical Engineering, University of New South Wales Sydney, Sydney, NSW 2052 Australia; 5 Cancer Research UK Beatson Institute, Glasgow Scotland, G61 BD UK; 6 Department of Physiology, Anatomy and Microbiology, School of Life Sciences, La Trobe University, Bundoora, VIC 3086 Australia; 7 Department of Biomedical Sciences, College of Veterinary Medicine, Cornell University, Ithaca, NY 14853 USA; 8 Department of Bioengineering, Institute of Engineering in Medicine, University of California, San Diego, CA 92121 USA; 9 Children’s Medical Research Institute, University of Sydney, Sydney, NSW 2006 Australia; 10 Department of Pathology, Dunedin School of Medicine, University of Otago, Dunedin, 9054 New Zealand; 11 Maurice Wilkins Centre, University of Otago, Dunedin, 9054 New Zealand; 12 Biomedical imaging facility, Lowy Cancer Research Centre, University of New South Wales, Sydney, NSW Australia; 13 Pancreatic Cancer Translational Research Group, Lowy Cancer Research Centre, School of Medical Sciences, University of New South Wales, Sydney, NSW 2052 Australia; 14 Australian Centre for Nanomedicine, University of New South Wales, Sydney, NSW 2052 Australia; 15 Sydney Medical School, University of Sydney, Sydney, NSW 2006 Australia; 16 NSW Health Pathology, Department of Anatomical Pathology, Royal North Shore Hospital, St Leonards, Sydney, NSW 2065 Australia; 17 Cancer Diagnosis and Pathology Research Group, Kolling Institute of Medical Research, St Leonards, NSW 2065 Australia; 18 Schools of Life and Environmental Sciences, the Charles Perkin Centre, the University of Sydney, Sydney, NSW 2006 Australia; 19 The Kinghorn Cancer Centre, Garvan Institute of Medical Research, 370 Victoria Street, Darlinghurst, Sydney, NSW 2010 Australia; 20 Wollongong Hospital, Illawarra and Shoalhaven Local Health District, Loftus Street, Wollongong, NSW 2500 Australia; 21 Royal North Shore Hospital, Westbourne Street, St Leonards, NSW 2065 Australia; 22 QIMR Berghofer Medical Research Institute, 300 Herston Rd, Herston, QLD 4006 Australia; 23 University of Melbourne, Centre for Cancer Research, Victorian Comprehensive Cancer Centre, 305 Grattan Street, Melbourne, VIC 3000 Australia; 24 Institute for Molecular Bioscience, University of QLD, St Lucia, QLD 4072 Australia; 25 Bankstown Hospital, Eldridge Road, Bankstown, NSW 2200 Australia; 26 Liverpool Hospital, Elizabeth Street, Liverpool, NSW 2170 Australia; 27 St Vincent’s Hospital, 390 Victoria Street, Darlinghurst, N W 2010 Australia; 28 Westmead Hospital, Hawkesbury and Darcy Roads, Westmead, NSW 2145 Australia; 29 Royal Prince Alfred Hospital, Missenden Road, Camperdown, NSW 2050 Australia; 30 Prince of Wales Hospital, Barker Street, Randwick, NSW 2031 Australia; 31 Fremantle Hospital, Alma Street, Fremantle, WA 6959 Australia; 32 Sir Charles Gairdner Hospital, Hospital Avenue, Nedlands, WA 6009 Australia; 33 St John of God Healthcare, 12 Salvado Road, Subiaco, WA 6008 Australia; 34 Royal Adelaide Hospital, North Terrace, Adelaide, SA 5000 Australia; 35 Flinders Medical Centre, Flinders Drive, Bedford Park, SA 5042 Australia; 36 Envoi Pathology, 1/49 Butterfield Street, Herston, QLD 4006 Australia; 37 Princess Alexandria Hospital, Cornwall Street & Ipswich Road, Woolloongabba, QLD 4102 Australia; 38 Austin Hospital, 145 Studley Road, Heidelberg, VIC 3084 Australia; 39 Johns Hopkins Medical Institute, 600 North Wolfe Street, Baltimore, MD 21287 USA; 40 ARC-NET Center for Applied Research on Cancer, University of Verona, Via dell’Artigliere, 19, Verona, 37129 Province of Verona Italy; 41 Wolfson Wohl Cancer Research Centre, Institute of Cancer Sciences, University of Glasgow, Garscube Estate, Switchback Road, Bearsden, Glasgow, Scotland, G61 1BD UK

**Keywords:** Cancer, Cancer microenvironment, Cell biology

## Abstract

Heterogeneous subtypes of cancer-associated fibroblasts (CAFs) coexist within pancreatic cancer tissues and can both promote and restrain disease progression. Here, we interrogate how cancer cells harboring distinct alterations in p53 manipulate CAFs. We reveal the existence of a p53-driven hierarchy, where cancer cells with a gain-of-function (GOF) mutant p53 educate a dominant population of CAFs that establish a pro-metastatic environment for GOF and null p53 cancer cells alike. We also demonstrate that CAFs educated by null p53 cancer cells may be reprogrammed by either GOF mutant p53 cells or their CAFs. We identify perlecan as a key component of this pro-metastatic environment. Using intravital imaging, we observe that these dominant CAFs delay cancer cell response to chemotherapy. Lastly, we reveal that depleting perlecan in the stroma combined with chemotherapy prolongs mouse survival, supporting it as a potential target for anti-stromal therapies in pancreatic cancer.

## Abstract (web summary)

Subtypes of cancer associated fibroblasts can both promote and suppress tumorigenesis. Here, the authors investigate how p53 status in pancreatic cancer cells affects their interaction with cancer associated fibroblasts, and report perlecan as a mediator of the pro-metastatic environment.

## Introduction

Pancreatic cancer (PC) is one of the largest causes of cancer-related death, with ~7% survival rate at 5 years post-diagnosis^1^. New therapeutics have been employed to improve patient outcomes; however, median survival remains less than 1 year^2,3^. Consequently, identifying new therapeutic targets is a critical need in this disease.

*TP53* is one of the most commonly altered genes in human cancer and changes in this gene correlate with poor patient outcomes in a broad range of tumor types including PC^4–9^. Wild-type (WT) *TP53* is a tumor-suppressor, which acts as the guardian of the genome to trigger DNA repair or elimination of damaged cells^10–12^. Mutations in *TP53* most commonly induce either a loss of WT function (null mutations) or acquisition of pro-tumorigenic functions (gain-of-function (GOF) mutations). Similarly, loss of p53 expression may also occur as a result of genetic aberrations^4^.

Genetically engineered mouse models (GEMMs) of PC have provided key insights into how alterations in *TP53* drive disease progression^13–15^. We and others have used the poorly-metastatic Pdx1-Cre; LSL-K-ras^G12D/+^; LSL-p53^fl/+^ (KPflC) and the highly metastatic Pdx1-Cre; LSL-K-ras^G12D/+^; LSL-p53^R172H/+^ (KPC) mouse models. These models differ only in the p53 status of pancreatic epithelial cells and previous work demonstrated that mutant p53^R172H^ in the epithelial cells drove the progression of invasive and metastatic PC over and above the effects of loss-of-p53^8,16–19^, in agreement with observations in patients^4^.

While the role of p53 mutations in driving cancer cell behavior has been extensively studied^4^, here we investigate whether alterations in p53 in pancreatic tumor cells trigger cell extrinsic effects and influence features of surrounding stromal cancer-associated fibroblasts (CAFs). Pancreatic tumors are commonly characterized by stromal remodeling^2,20,21^, which leads to altered interactions between cancer cells and their surrounding environment. We and others have previously demonstrated that re-shaping the pancreatic stroma can impair tumor progression and improve the efficacy of standard-of-care therapies^2,22–27^. This feedback is reciprocal, and cancer cells with distinct characteristics have been shown to locally tune the tumor stroma^28–30^. For instance CAFs, which are recruited and activated during cancer development^30,31^, play a critical role in establishing a protective and pro-tumorigenic environment^2,32^, and as such represent an attractive therapeutic target. However, chronic ablation of CAFs in pancreatic tumors has yielded distinctly mixed results, highlighting how CAFs can both promote and impair cancer progression^28,32–36^. In addition, it has become clear that not all CAFs are alike; for example, spatially distinct populations of CAFs were shown to play different roles in cancer progression^30^. Consequently, understanding the intricate, cell-specific crosstalk occurring between distinct cancer and stromal cell populations will be critical to develop effective stromal-based therapeutics for PC, which is a highly molecularly heterogeneous disease^5,9,30,37,38^.

In this study, we characterize CAFs isolated from KPflC (p53 null) and KPC (GOF mutant p53) end-stage primary tumors. We probe short and long-range interactions between the cancer cells and their CAFs in both tumor types to interrogate how CAF reprogramming by the respective KPflC and KPC cancer cells regulate PC progression. We unravel how cells with different p53 status can influence one another to modulate invasion, metastasis and response to chemotherapy. We also present evidence that manipulating the stromal deposition of perlecan (heparin sulphate proteoglycan 2, HSPG2) may offer future therapeutic opportunities in this devastating disease.

## Results

### Isolation and characterization of primary CAFs

**Fig. 1:**
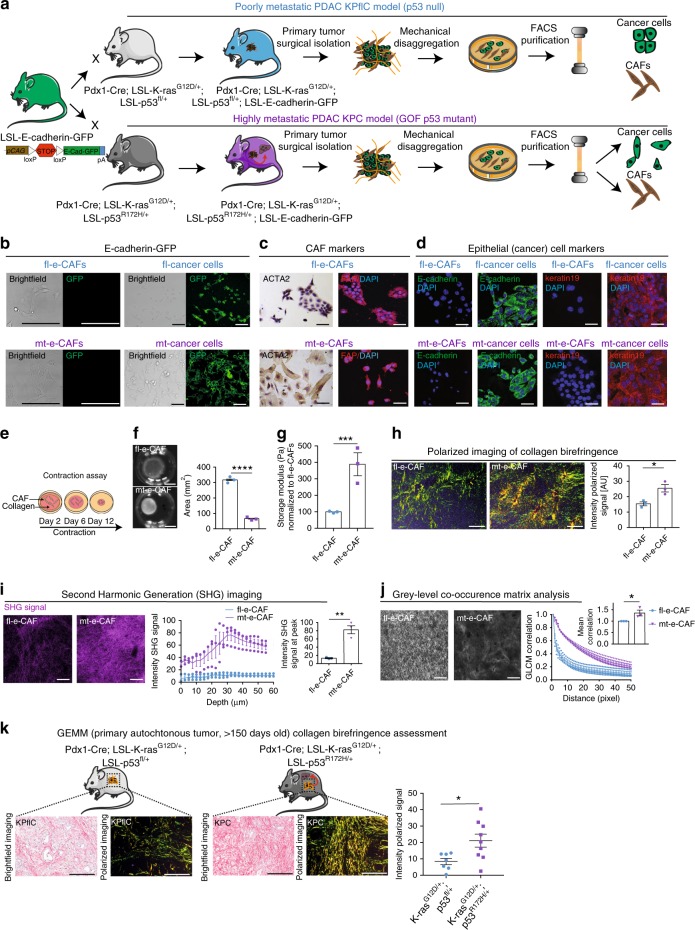
Isolation and characterization of CAFs from KPflC and KPC primary tumors. **a** Representation of isolation of cancer cells and CAFs from poorly metastatic KPflC (top panel) and highly metastatic KPC (lower panel) primary tumors using the previously generated LSL-E-cadherin-GFP mouse. **b** Brightfield and GFP imaging of cancer cells and CAFs isolated from E-Cadherin-GFP-KPflC tumors and from E-Cadherin-GFP-KPC tumors. Scale bar: 100 μm. **c** Immunocytochemistry staining of markers of CAF activation (ACTA2 α-smooth muscle actin; FAP fibroblast activated protein) in fl-e-CAFs and mt-e-CAFs. Scale bar: 100 μm. **d** Immunofluorescence staining of CAFs and cancer cells for markers of pancreatic epithelial cells (E-cadherin, keratin 19). Scale bar: 100 μm. **e** Representation of CAF-driven contraction assay. **f** Representative images of CAF-collagen matrices and quantification of matrix area following a 12-day contraction assay. Scale bar: 1 cm, *n* = 3 biological repeats with three technical replicates per biological repeat. **g** Shear rheology measurements of storage modulus of matrices remodeled by fl-e-CAFs or by mt-e-CAFs and normalized to values found in fl-e-CAFs matrices. *n* = 3 biological repeats with three technical replicates per biological repeat. **h** Polarized light imaging and quantification of picrosirius red-stained collagen matrices remodeled by fl-e-CAFs or by mt-e-CAFs. Scale bar: 100 μm, *n* = 3 biological repeats with three technical replicates per biological repeat. **i** Representative maximum projections of Second Harmonic Generation (SHG) signal in collagen matrices remodeled by fl-e-CAFs and by mt-e-CAFs and quantification of SHG signal intensity through a 60 μm z-stack and at intensity peak (bar graph inset). Scale bar: 100 μm, *n* = 3 biological repeats with three technical replicates per biological repeat. **j** Representative single-plane SHG images of collagen matrices generated by fl-e-CAFs or by mt-e-CAFs and quantification of GLCM correlation (bar graph inset depicts GLCM mean correlation normalized to mean correlation found in fl-e-CAFs matrices). Scale bar: 100 μm, *n* = 3 biological repeats with three technical replicates per biological repeat. **k** Representative images of picrosirius red staining imaged with brightfield or polarized light and quantification of collagen birefringence signal in primary KPflC and KPC tumors. *n* = 7 for KPflC tumors and *n* = 9 for KPC tumors. Data are presented as mean values with SEM. *p < 0.05, **p < 0.01, ***p < 0.001, ****p < 0.0001

CAFs were isolated from primary tumors developed in the poorly-metastatic KPflC GEMM (Fig. 1a top panel) or in the highly metastatic KPC GEMM (Fig. 1a lower panel). KPflC and KPC mice were crossed with the LSL-E-cadherin-GFP reporter mouse, where epithelial cancer cells express GFP at the cell-cell junction while matched CAFs from the respective models do not^39^. Once autochthonous PDAC developed fully (mice > 150 days old), CAFs were isolated by mechanical disaggregation of the primary tumor and fluorescence activated cell sorting (FACS, Fig. 1a and Methods section)^29,40^. Following FACS isolation, imaging of GFP in cancer cells and matched CAFs confirmed that CAFs isolated from both GEMMs do not express E-cadherin-GFP (Fig. 1b), while immunohistochemistry (IHC) and immunofluorescence analyses of markers for CAFs^29^ (Fig. 1c) and pancreatic epithelial cancer cells (Fig. 1d) validated the identity and purity of the isolated CAFs. CAF activation was confirmed using an in vitro collagen contraction assay (Fig. 1e and Supplementary Fig. 1a)^22,39^. While both CAF populations were able to actively remodel collagen, there was a greater degree of contraction in CAF matrices isolated from KPC tumor compared to KPflC tumor (hereafter referred to as mutant-educated CAFs, or mt-e-CAFs and as flox-educated-CAFs, or fl-e-CAFs, respectively (Fig. 1f and Supplementary Fig. 1a)). Further, markers of CAF contractility (pMLC, pMYPT1, and ACTA2^22,41^) were found to be expressed at a higher level in mt-e-CAFs compared to fl-e-CAFs (Supplementary Fig. 1b). We observed that the expression of FAP, a marker of CAF activation^37,42^, was higher in mt-e-CAFs compared to fl-e-CAFs when embedded in a collagen matrix but not when cultured in 2D (Supplementary Fig. 1b, c), suggesting a context-specific difference in activation between the CAF populations.

We then assessed the properties of collagen matrices remodeled by fl-e-CAFs and by mt-e-CAFs. Using shear rheology analysis and atomic force microscopy, we found that mt-e-CAFs form mechanically stiffer matrices than fl-e-CAFs (Fig. 1g and Supplementary Fig. 1d, e). Polarized imaging of picrosirius red staining^22,43^ in CAF-collagen matrices demonstrated a significant increase of fibrillar collagen density in matrices remodeled by mt-e-CAFs compared to matrices established by fl-e-CAFs (Fig. 1h). This was confirmed by Second Harmonic Generation (SHG) imaging (Fig. 1i) and via analysis of collagen fiber ultrastructure using grey-level co-occurrence matrix (GLCM) assessment (Fig. 1j and Supplementary Fig. 1f)^22,25,44^. Similarly, we found higher levels of fibrillar collagen in autochthonous primary tumors in highly-metastatic KPC tumors compared to poorly-metastatic KPflC tumors (Fig. 1k), demonstrating that the isolated CAFs recapitulate critical features of the ECM found in native pancreatic tumor tissues. The phenotype of CAFs can be altered during in vitro culture and we performed contraction assays to compare the properties of CAFs after FACS isolation (early) with those that have been subjected to 10 population doublings (late). We found that mt-e-CAFs consistently form smaller matrices with higher fibrillar collagen content compared to fl-e-CAFs, both at early and late passages (Supplementary Fig. 1g, h), demonstrating that the properties of the isolated CAFs persist following in vivo education. For further experiments, we worked with early passages of both CAFs, to avoid the possibility of spontaneous immortalization.

### fl-e-CAFs can be further educated by mt-cells

**Fig. 2:**
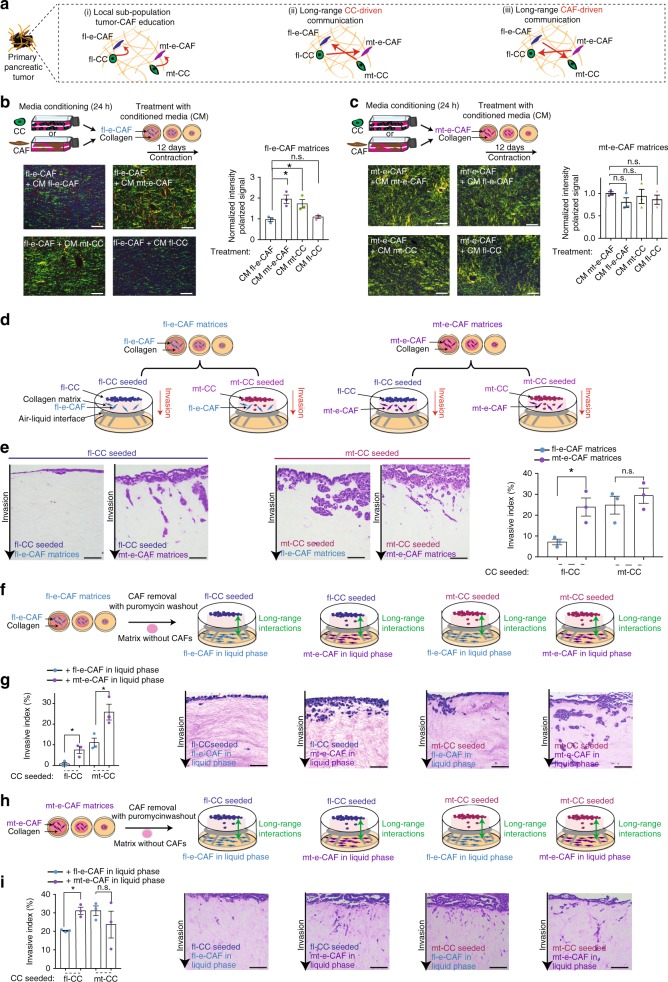
mt-cells further potentiate fl-e-CAFs and mt-e-CAFs to induce fl-CCs invasion. **a** Representation of (i) local crosstalk between cancer cells and CAFs, (ii) long-range CC driven interactions, and (iii) long-range CAF-driven interactions between cancer cells and CAFs across a primary pancreatic tumor. **b** Top panel: representation of media conditioning driven by cancer cells or by CAFs and treatment of fl-e-CAFs with conditioned media (CM) during remodeling of a collagen matrix over 12 days. Lower panel: representative images and quantification of collagen birefringence in fl-e-CAFs matrices treated with CM generated by fl-e-CAFs, mt-e-CAFs, mt-CCs or fl-CCs, and normalized to treatment with CM from fl-e-CAFs. Scale bar: 100 μm. **c** Top panel: representation of media conditioning driven by cancer cells or by CAFs and treatment of mt-e-CAFs with CM during remodeling of a collagen matrix over 12 days. Lower panel: representative images and quantification of collagen birefringence in mt-e-CAFs matrices treated with CM generated by mt-e-CAFs, fl-e-CAFs, mt-CCs or fl-CCs, and normalized to treatment with CM from mt-e-CAFs. Scale bar: 100 μm. **d** Schematic representation of 3D CAF-based organotypic invasion assay. **e** Representative H&E staining of organotypic invasion assay and quantification of invasive index for fl-CCs or mt-CCs invading into fl-e-CAF matrices (blue bars) or into mt-e-CAFs matrices (purple bars). Scale bar: 200 μm. **f** Schematic representation of 3D organotypic invasion assay including pharmaceutical removal of CAFs following matrix establishment and continuous media conditioning driven by CAFs during cancer cell invasion. **g** Quantification of invasive index and representative images of H&E stained organotypic matrices with fl-CCs and mt-CCs invading into matrices generated by fl-e-CAFs and with continuous media conditioning driven by fl-e-CAFs or by mt-e-CAFs during invasion. Scale bar: 200 μm. **h** Schematic representation, **i** quantification of invasive index and representative images of H&E stained organotypic matrices with fl-CCs and mt-CCs invading into matrices generated by mt-e-CAFs and with continuous media conditioning driven by fl-e-CAFs or by mt-e-CAFs during cancer cell invasion. Scale bar: 200 μm. Contraction and invasion assays were conducted in three biological repeats with three technical replicates per biological repeat. Individual data points are presented with mean values and SEM. *p < 0.05

We and others previously demonstrated that PC is a molecularly heterogeneous disease^5,9,30,37,38^, where spatially distinct regions of the tumor tissue can host different cancer cell and CAF populations that may interact via direct, local or long-range communication within the tumor tissue (Fig. 2a). As such, cancer cells harboring a GOF mutant p53 (hereafter referred to as mt-CCs) could co-opt and activate local fibroblasts, while in a spatially distinct region of the tumor tissue, cancer cells with a loss of p53 (herein referred to as fl-CCs), which are less invasive, can recruit and shape different CAF populations^45–47^(Fig. 2a (i)). To ask whether signaling across the tumor tissue also exists between these subpopulations (Fig. 2a (ii, iii)), we monitored how direct and long-range interactions between cancer cells and CAFs isolated from the poorly-metastatic and highly-metastatic GEMMs affect key events of cancer progression.

To mimic long-range interactions between CC and CAF populations during ECM remodeling, we treated contraction assays of fl-e-CAFs (Fig. 2b) and mt-e-CAFs (Fig. 2c) with conditioned media (CM) generated by fl-e-CAFs, mt-e-CAFs, fl-CCs, or mt-CCs. fl-e-CAF matrices demonstrated an increase in fibrillar collagen density upon treatment with CM generated by mt-e-CAFs or by mt-CCs compared to CM generated by fl-e-CAFs or by fl-CCs (Fig. 2b). Conversely, treating mt-e-CAF matrices with CM generated by fl-e-CAFs, fl-CCs or mt-CCs did not significantly alter collagen birefringence compared to treatment with CM generated by mt-e-CAFs (Fig. 2c). This suggests that factors secreted by mt-e-CAFs or by mt-CCs can potentiate fl-e-CAFs to increase matrix remodeling.

As cancer cell invasion can be regulated by the properties of the surrounding ECM as well as by CAF-derived signaling^26,48–51^, we interrogated how cancer cell invasion can be influenced by direct and long-range interactions between the four cell subtypes^22,39,43^.

### mt-e-CAFs create a permissive environment for invasion

fl-CCs and mt-CCs were seeded onto matrices generated by either fl-e-CAFs or mt-e-CAFs and allowed to invade (Fig. 2d). p53 status in fl-CCs and mt-CCs was confirmed using IHC staining (Supplementary Fig. 2a). In line with previous studies in vivo^16,18^, fl-CCs invaded poorly into matrices remodeled by their matched fl-e-CAFs (Fig. 2d, e), while mt-CCs were highly invasive in matrices generated by their matched mt-e-CAFs. Interestingly, fl-CCs were able to invade into matrices remodeled by mt-e-CAFs to a similar degree as mt-CCs (Fig. 2d, e), while mt-CCs invaded independently of the CAF-driven matrix remodeling (Fig. 2d, e). Similar changes in cell invasiveness were found using fl-CCs transfected with either a control empty vector (EV) or with the human equivalent of murine p53^R172H^ (p53^R175H^, Supplementary Fig. 2b, c^18,19,52,53^). This suggests that CAFs educated by cancer cells with a GOF mutant p53 provide cancer cells with pro-invasive cues that are not present in fl-e-CAF matrices. The enhanced ability of fl-CCs to invade into matrices generated by mt-e-CAFs could potentially be due to the increased stiffness found in these matrices (Fig. 1g and Supplementary Fig. 1d, e), in line with previous work^49^.

To dissect how direct contact between CAFs and CCs influences cancer cell motility, we imaged the interactions between t-RFP labeled CAFs and GFP labeled CCs co-seeded onto a cell-derived matrix (CDM, Supplementary Fig. 2d^22,54^). We quantified coordinated cell movement, or streaming, using image anisotropy^22,55^. Similarly to Fig. 2d, e, fl-CC collective migration was enhanced in the presence of mt-e-CAFs compared to fl-e-CAFs (Supplementary Fig. 2e). In contrast, mt-CC streaming was not significantly different in the presence of mt-e-CAFs versus fl-e-CAFs (Supplementary Fig. 2f). We observed three distinct classes of dynamic CAF-CC interactions on CDMs: (i) static interactions where t-RFP-CAFs and GFP-CCs remain in close proximity without migrating (Supplementary Fig. 2g (i)); (ii) CAF-driven migration where movement of a group of CCs is directed by the CAFs (Supplementary Fig. 2g (ii) and (iii)) and parallel migration where CAFs and CCs stream together in a parallel direction (Supplementary Fig. 2g (iii)). Scoring of these interactions revealed that fl-e-CAFs and fl-CCs mainly follow the static interactions, while fl-CCs seeded with mt-e-CAFs use CAF-driven migration and parallel migration during streaming (Supplementary Fig. 2h). This suggests that mt-e-CAFs directly interact with fl-CCs to promote their collective movement, partially explaining the effects observed in 3D matrices (Fig. 2d, e), and in line with previous work^48,56^. Conversely, mt-CCs streaming in the presence of their corresponding mt-e-CAFs relied primarily on CAF-driven migration and parallel migration, while static interactions only occurred in the presence of fl-e-CAFs (Supplementary Fig. 2h).

We next interrogated whether long-range interactions between CAFs and CCs can also influence cancer cell invasion. Collagen matrices were generated using fl-e-CAFs and mt-e-CAFs as before, after which CAFs were pharmacologically removed (Fig. 2f, h). CCs were then seeded and allowed to invade into the collagen matrix, while fl-e-CAFs or mt-e-CAFs were seeded under the metal grid in order to expose invading CCs to media continuously conditioned by CAFs (Fig. 2f, h). Invasion of fl-CCs or mt-CCs into matrices originally made using fl-e-CAFs that were subsequently removed, was increased upon exposure to media continually conditioned by mt-e-CAFs compared to fl-e-CAFs (Fig. 2g and Supplementary Fig. 2i with DMEM control). On matrices originating from mt-e-CAFs that have subsequently been removed, media conditioned by mt-e-CAFs also increased fl-CC invasion (Fig. 2i); however, it did not significantly alter mt-CC invasion (Fig. 2i). This suggests that mt-e-CAFs can also signal to fl-CCs in a long-range manner to render them more invasive in both poorly remodeled and more permissive collagen matrices.

While GOF mutant p53 in the cancer cell compartment has previously been shown to drive a pro-invasive program^4,8,17,18,52,53,57,^, our results using long-range conditioning, CAF-based organotypic assays and cell streaming experiments reveal that mt-e-CAFs can induce fl-CC invasion by creating an environment more favorable to invasion, via direct contact and through long-range, paracrine interactions.

### mt-CCs support fl-CC invasion through less-permissive environments

**Fig. 3:**
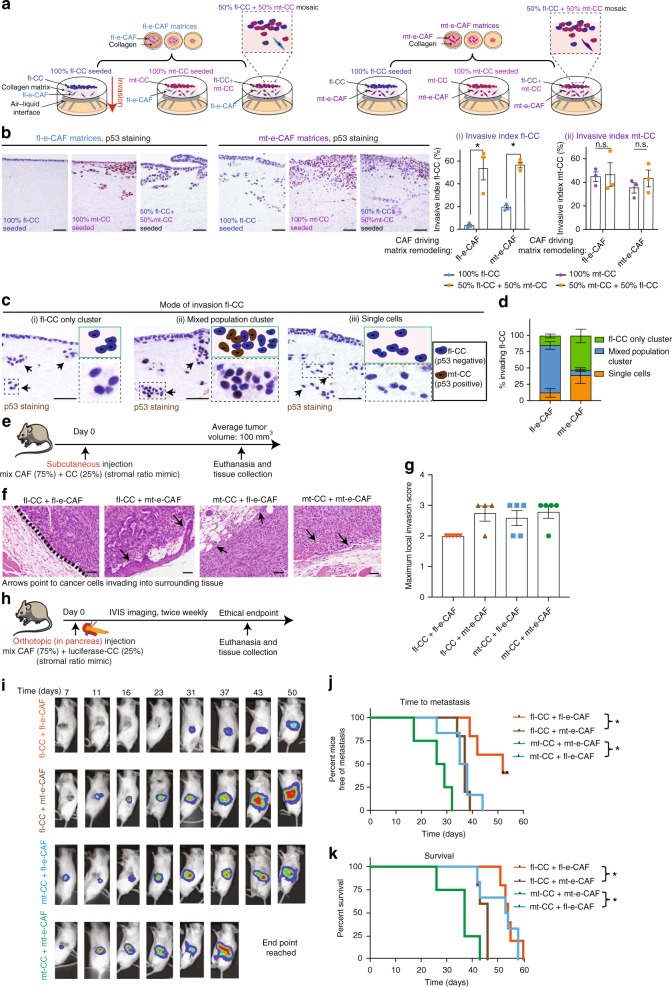
mt-CCs act as carrier for fl-CCs and mt-e-CAFs drive local invasion and metastasis in vivo. **a** Schematic representation of 3D organotypic invasion assays with mosaic cancer cell cultures invading into matrices generated by fl-e-CAFs or mt-e-CAFs, **b** representative images of p53 staining (PAb421 antibody) and quantification of invasive index of (i) fl-CCs and (ii) mt-CCs invading alone or in a mosaic culture (fl-CCs + mt-CCs 50:50 ratio) into fl-e-CAF matrices or into mt-e-CAF matrices. Scale bar: 200 μm, *n* = 3 biological repeats with three technical replicates per biological repeat. **c** Representative images and schematic representation of p53 staining (PAb421 antibody) in organotypic matrices and depicting fl-CCs invading either as a cluster of fl-CCs only (i), as a mosaic cluster with mt-CCs (ii) or as single fl-CCs (iii). Scale bar: 200 μm. **d** Quantification of the mode of invasion followed by fl-CCs during invasion into fl-e-CAF matrices or into mt-e-CAF matrices and in the presence or absence of mt-CCs, *n* = 3 biological repeats with three technical replicates per repeat. **e** Schematic representation of subcutaneous injection of cancer cells (25%) with CAFs (75%), tumor growth and mouse euthanasia followed by tissue collection. **f** Representative H&E images, and **g** quantification of mean maximum score of cancer cell local invasion into the surrounding tissue in subcutaneous xenografts generated by fl-CCs injected with fl-e-CAFs (orange dots, *n* = 5 mice) or injected with mt-e-CAFs (brown dots, *n* = 4 mice) and in subcutaneous xenografts generated by mt-CCs injected with fl-e-CAFs (blue dots, *n* = 5 mice) or with mt-e-CAFs (green dots, n = 5 mice). Scale bar: 200 μm. **h** Schematic representation of orthotopic (in pancreas) injection of 50 luciferase-expressing CCs (fl-CCs or mt-CCs) with 150 CAFs (fl-e-CAFs or mt-e-CAFs) and IVIS whole body monitoring of tumor growth and metastatic spread. **i** Representative images of whole body IVIS imaging in mice bearing orthotopic xenografts, and **j** Kaplan–Meier survival analysis of time to metastasis and of **k** survival of mice orthotopically injected with fl-CCs + fl-e-CAFs (*n* = 5 mice); fl-CCs + mt-e-CAFs (*n* = 5 mice); mt-CCs + fl-e-CAFs (*n* = 6 mice) or with mt-CCs + mt-e-CAFs (*n* = 4 mice). Individual data points are presented with mean values and SEM. *p < 0.05

Given the heterogeneity found in PC cells^5,9^, we next sought to assess how fl-CCs and mt-CCs, which can both reside in the same tumor^37,58^, may influence the invasive behavior of one another. We allowed fl-CCs alone, mt-CCs alone or a mosaic mix of fl-CCs and mt-CCs (1:1 ratio) to invade into collagen matrices originally created by fl-e-CAFs or by mt-e-CAFs (Fig. 3a). We observed that in matrices generated by fl-e-CAFs or by mt-e-CAFs, invasion of fl-CCs was increased in the presence of mt-CCs compared to fl-CCs alone (Fig. 3b (i)). mt-CC invasion was not significantly affected by the presence of fl-CCs in either type of matrix (Fig. 3b (ii)).

We also observed that fl-CCs followed three distinct modes of invasion; (i) as fl-CC-only clusters (Fig. 3c (i)), (ii) fl-CCs in clusters with mt-CCs (Fig. 3c (ii) or (iii) as single cells (Fig. 3c (iii)). Scoring of these distinct patterns of invasion demonstrated that in non-permissive fl-e-CAF matrices, fl-CCs most notably rely on forming clusters with mt-CCs to invade, while they predominantly invaded as single cells when seeded on a permissive mt-e-CAF matrix (Fig. 3d). This suggests that in non-permissive matrices (Fig. 2d, e), mt-CCs can act as carriers for the otherwise poorly invasive fl-CCs. However, fl-CCs do not need to interact with mt-CCs when invading into the invasion-permissive matrices remodeled by mt-e-CAFs.

Guided by the observed increase of cancer cell invasion upon interactions with mt-e-CAFs, we investigated how CC-CAF crosstalk influences cancer cell invasive behaviors in vivo.

### CAF-cancer cell crosstalk drives invasion and metastasis in vivo

fl-CCs or mt-CCs were subcutaneously injected with either fl-e-CAFs or mt-e-CAFs into the rear flank of Balb/c-Fox1nuAusb mice (Fig. 3e). A 1:3 ratio of CC:CAF was used to mimic the high stromal content and low tumor cellularity commonly found in PDAC^25^. Co-injection of either fl-e-CAFs or mt-e-CAFs with fl-CCs or mt-CCs did not significantly affect tumor growth (Supplementary Fig. 3a). Analysis of local invasion revealed that fl-CCs co-injected with fl-e-CAFs formed encapsulated tumors with well-defined margins; while fl-CCs and mt-CCs co-injected with mt-e-CAFs displayed increased invasion into surrounding tissue (Fig. 3f, g, Supplementary Fig. 3b and Methods for details on scoring of local invasion^25^). mt-CCs readily invaded into the surrounding tissue when injected with fl-e-CAFs or mt-e-CAFs (Fig. 3f, g). In agreement with our findings in contraction assays and in autochthonous pancreatic tumors (Fig. 1h, k), we also observed an increase of collagen birefringence in tumors generated by fl-CCs and mt-CCs co-injected with mt-e-CAFs compared to co-injection with fl-e-CAFs (Supplementary Fig. 3c). Given that ECM remodeling plays a critical role in driving cancer cell motility^2,22,49,59^, this may partially explain the enhanced invasiveness of fl-CCs and mt-CCs in the presence of mt-e-CAF compared to fl-e-CAF.

Next, the long-term effects of interactions between CAFs and cancer cells on metastasis and survival were assessed using orthotopic co-injections of cancer cells expressing luciferase with CAFs expressing t-RFP into the pancreas of NOD/SCID/ILR2γ^−/−^ mice (Fig. 3h). Tumor progression was monitored via twice-weekly IVIS Luciferase imaging^22,60^. Metastatic burden and occurrence of ascites were increased (percent of mice with ascites at endpoint: fl-CCs + fl-e-CAFs: 0%, fl-CCs + mt-e-CAFs: 40%, mt-CCs + fl-e-CAFs: 50%, and mt-CCs + mt-e-CAFs: 100%), and survival was reduced when fl-CCs or mt-CCs were co-injected with mt-e-CAFs compared to fl-e-CAFs (Fig. 3i–k), indicating that mt-e-CAFs facilitate metastatic spread for both fl-CCs and mt-CCs. Staining for t-RFP in tumors at endpoint demonstrated that both fl-e-CAFs and mt-e-CAFs are maintained (Supplementary Fig. 4a), highlighting their continued role during cancer progression in this setting. In addition, metastatic burden in the liver and the lungs at endpoint was enhanced for fl-CCs co-injected with mt-e-CAFs compared to fl-e-CAFs and was not significantly different for mt-CCs when co-injected with either fl-e-CAFs or mt-e-CAFs (Supplementary Fig. 4b, c). Lastly, we confirmed that the increase of metastatic burden and the reduced survival were not a result of changes in the proliferation rate of cancer cells upon co-injection with CAFs by quantifying the in vivo luciferase signal at the early stages of cancer progression (Fig. 3h–k and Supplementary Fig. 4d) and by measuring the in vitro doubling time of cancer cells co-cultured with fl-e-CAFs or mt-e-CAFs (Supplementary Fig. 4e).

In order to identify factors potentially responsible for the enhanced invasion and metastatic spread observed in the presence of mt-e-CAFs, we next profiled the secretome of CAFs isolated from the poorly-metastatic KPflC and the highly-metastatic KPC models.

### HSPG2 is secreted by mt-e-CAFs and drives metastasis

**Fig. 4:**
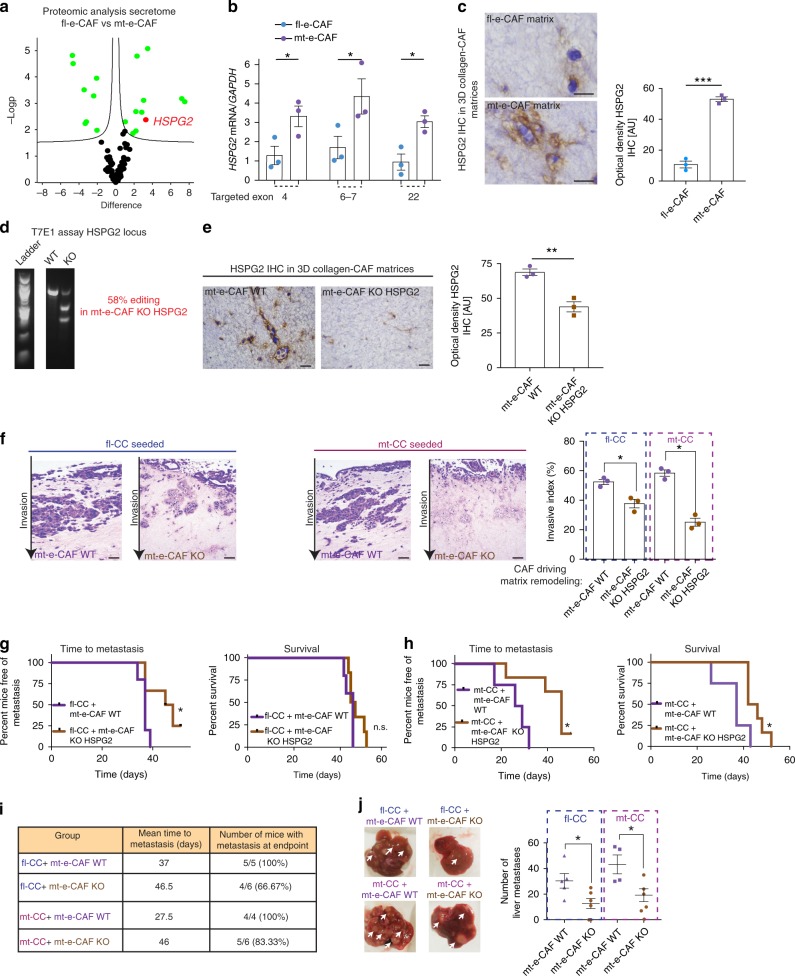
HSPG2 is a critical component of the permissive environment created by mt-e-CAFs. **a** Volcano plot depicting proteins differently secreted by fl-e-CAFs (left) and by mt-e-CAFs (right), *n* = 5 biological repeats. **b**. RT-qPCR analysis of HSPG2 mRNA levels in fl-e-CAFs and mt-e-CAFs, *n* = 3 biological repeats. **c** HSPG2 IHC staining and quantification of optical density of staining in collagen matrices remodeled by fl-e-CAFs and by mt-e-CAFs. Scale bar: 25 μm, *n* = 3 biological repeats with three technical replicates per biological repeat. **d** T7E1 assay at the HSPG2 locus in mt-e-CAFs WT and in a mixed population of mt-e-CAFs KO HSPG2. **e** HSPG2 IHC staining and quantification of optical density in collagen matrices remodeled by mt-e-CAFs WT or mt-e-CAFs KO HSPG2. Scale bar: 100 μm, *n* = 3 biological repeats with three technical replicates per biological repeat. **f** Representative H&E stained sections and quantification of invasive index for fl-CCs and mt-CCs invading into matrices remodeled by mt-e-CAFs WT or by mt-e-CAFs KO HSPG2. *n* = 3 biological repeats with three technical replicates per biological repeat. Scale bar: 100 μm. **g** Kaplan–Meier survival analysis of time to metastasis and Kaplan–Meier analysis of survival in mice orthotopically injected with fl-CCs + mt-e-CAFs WT (*n* = 5 mice, data shown in Fig. 3j-k) or with fl-CCs + mt-e-CAFs KO HSPG2 (*n* = 6 mice). **h** Kaplan–Meier survival analysis of time to metastasis and Kaplan–Meier analysis of survival in mice orthotopically injected with mt-CCs + mt-e-CAFs WT (*n* = 4 mice, data shown in Fig. 3j-k) or with fl-CCs + mt-e-CAFs KO HSPG2 (*n* = 6 mice). **i** Table summarizing mean time to metastasis and mice with metastasis at endpoint, and **j** number of liver metastases for mice orthotopically injected with fl-CCs + mt-e-CAFs WT, fl-CCs + mt-e-CAFs KO HSPG2, mt-CCs + mt-e-CAFs WT or mt-CCs + mt-e-CAFs KO HSPG2. Individual data points are presented with mean values and SEM. *p < 0.05, **p < 0.01, ***p < 0.001

Mass-spectrometry analysis of the CAF secretomes using CM identified 19 proteins classed as secreted or extracellular proteins with significantly different levels of expression between fl-e-CAFs and mt-e-CAFs (Fig. 4a, Supplementary Fig. 5a and Supplementary Data 1). In particular, secretion of heparan sulphate proteoglycan 2 (HSPG2, or perlecan) was found to be increased in mt-e-CAF CM compared to fl-e-CAF CM, and this was confirmed using RT-qPCR and IHC staining in CAF-collagen matrices using both early and late passages of CAFs (Fig. 4b, c and Supplementary Fig. 5b). We therefore investigated the role of HSPG2 in establishing the pro-invasive and pro-metastatic environment created by mt-e-CAFs. HSPG2 is a multifunctional proteoglycan found in the ECM of most organs^61^. In prostate and oral squamous cancer, HSPG2 can play a role in fibrosis, cancer cell growth, angiogenesis, cell invasion and response to anti-cancer agents^62^; however, its role in PC has not been explored so far.

In order to evaluate the functional role of mt-e-CAF-derived HSPG2, we used CRISPR-Cas9 to knockout (KO) HSPG2 in mt-e-CAFs^63^. The efficacy of CRISPR-Cas9 editing of HSPG2 in a mixed population of mt-e-CAFs KO HSPG2 was confirmed using a T7 endonuclease 1 (T7E1) assay (Fig. 4d) and via IHC analyses (Fig. 4e). We initially used the mixed population of mt-e-CAFs KO HSPG2 to assess the effect of partial reduction of HSPG2 deposition on cancer cell invasion. fl-CCs and mt-CCs were seeded onto matrices generated by mt-e-CAFs WT or by a mixed population of mt-e-CAFs KO HSPG2 and allowed to invade. As before, both fl-CCs and mt-CCs readily invaded into mt-e-CAFs WT remodeled matrices (Fig. 4f, purple bars). In matrices remodeled by the mixed population of mt-e-CAFs KO HSPG2 we observed a reduction in invasion by fl-CCs and a larger reduction in invasion by mt-CCs (Fig. 4f, brown bars). Next, we injected fl-CCs or mt-CCs with mt-e-CAFs KO HSPG2 orthotopically (Fig. 4g). We compared metastatic burden and survival to the original orthotopic model using mt-e-CAFs WT (Fig. 3h–k). This revealed that survival was enhanced and metastatic burden was reduced in the presence of mt-e-CAFs KO HSPG2 compared to mt-e-CAFs WT for both fl-CCs and mt-CCs, with a more pronounced effect for mt-CCs (Fig. 4g–i). Metastatic burden in the liver at endpoint was reduced for both cancer cell types injected with mt-e-CAFs KO HSPG2 compared to mt-e-CAFs WT (Fig. 4j), while mice injected with mt-CCs also developed lung metastases (Supplementary Fig. 5c). The observed reduction of metastatic burden upon loss of stromal HSPG2 was not due to initial changes in cancer cell proliferation, as assessed by quantifying the in vivo luciferase signal and the in vitro doubling time of cancer cells co-cultured with CAFs (Supplementary Fig. 5d, e).

**Fig. 5:**
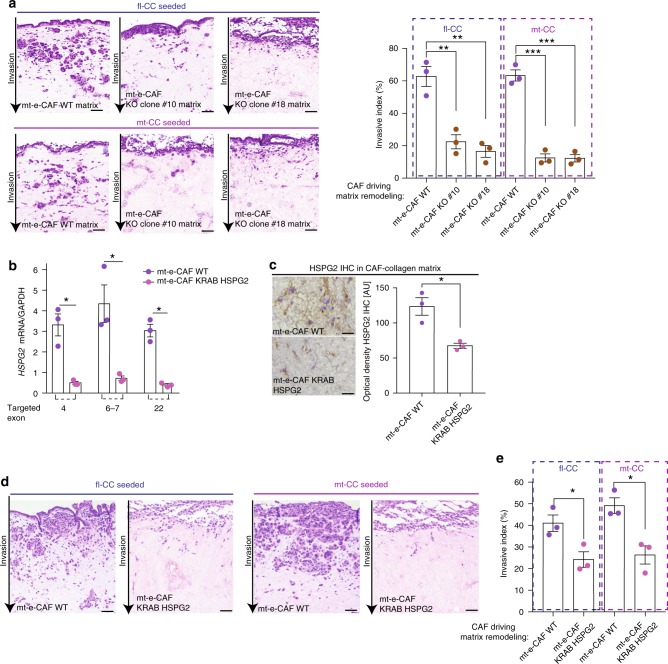
Depleting HSPG2 deposition reduces cancer cell invasion. **a** Representative H&E images and quantification of invasive index for fl-CCs and for mt-CCs invading into matrices remodeled by mt-e-CAFs WT or by clones 10 and 18 isolated from the mixed population of mt-e-CAFs KO HSPG2. Scale bar: 100 μm. **b** RT-qPCR analysis of HSGP2 mRNA in mt-e-CAFs WT and in mt-e-CAFs transfected with a KRAB HSPG2 construct. **c** HSPG2 IHC staining and quantification of optical density in collagen matrices generated by mt-e-CAFs WT or by mt-e-CAFs transfected with the KRAB HSPG2 construct. Scale bar: 100 μm. **d** Representative H&E images and **e** quantification of invasive index for fl-CCs and for mt-CCs invading into collagen matrices generated by mt-e-CAFs WT or by mt-e-CAFs transfected with a KRAB HSPG2 construct. Scale bar: 100 μm. All experiments were conducted with three biological repeats and with three technical replicates per biological repeat. Individual data points are presented with mean values and SEM. *p < 0.05, **p < 0.01, ***p < 0.001

We next isolated clonal populations from the mt-e-CAF KO HSPG2 population, and complete loss of HSPG2 was confirmed in two clones, #10 and #18, via genomic sequencing and IHC (Supplementary Fig. 6a-c). Invasion of both fl-CCs and mt-CCs into matrices remodeled by mt-e-CAF KO HSPG2 clones #10 and #18 was significantly reduced compared to mt-e-CAF WT matrices (Fig. 5a). Importantly, the reduction of cell invasion in matrices generated by mt-e-CAF KO HSPG2 clones #10 and #18 was more severe than in matrices generated by the mixed mt-e-CAF KO HSPG2 population (Fig. 4f versus Fig. 5a), demonstrating a dose-dependent effect of stromal HSPG2 manipulation on cancer cell invasion.

Lastly, we used CRISPR interference (KRAB-dCas9) to repress HSPG2 expression in the mt-e-CAFs. Reduced HSPG2 expression in mt-e-CAFs was confirmed via RT-qPCR and IHC analyses (Fig. 5b, c). The ability of fl-CCs and mt-CCs to invade into matrices with reduced HSPG2 deposition was decreased compared to invasion into matrices remodeled by mt-e-CAFs WT (Fig. 5d, e), thereby validating our initial finding (Fig. 4f and Fig. 5a).

While mt-CCs can readily invade into less-permissive matrices established by fl-e-CAFs (Fig. 2d, e), we found a reduction of mt-CC invasion when seeded onto matrices generated by mt-e-CAFs KO HSPG2 (Figs. 4f and 5a). It is possible that mt-CCs can educate fl-e-CAFs to secrete more HSPG2 during invasion assays (12 days), while modulation of HSPG2 cannot be achieved with CAFs that have been genetically engineered to suppress HSPG2 expression.

Collectively, we demonstrate that enhanced HSPG2 secretion by mt-e-CAFs is a key driver of the establishment of an environment permissive to cell invasion and metastasis in both in vitro and in vivo models.

### Paracrine signaling mediated by NFκB induces HSPG2 expression

**Fig. 6:**
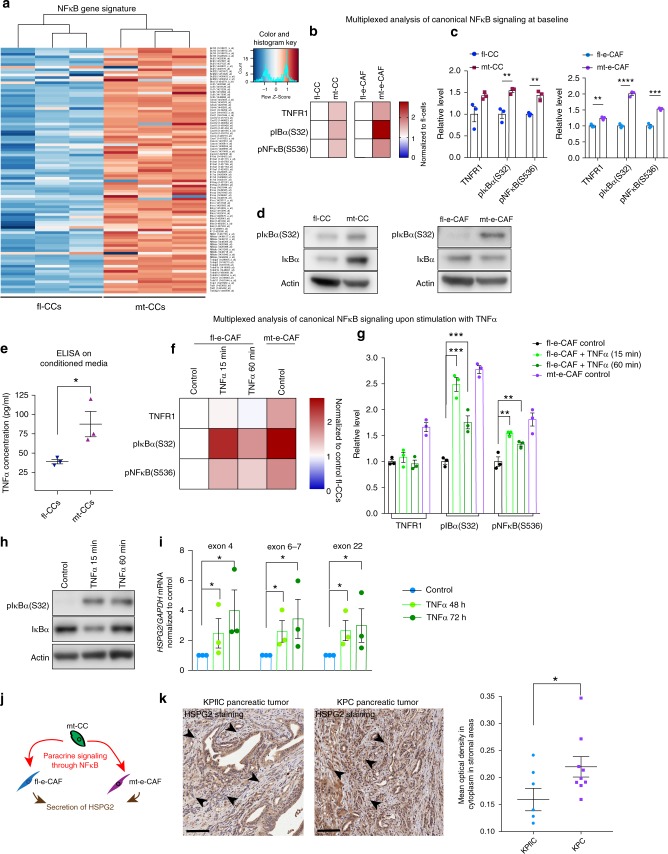
Paracrine signaling between mt-CCs and CAFs mediated by NFκB induces HSPG2 expression. **a** Heat map of microarray analysis of NFκB-target genes in fl-CCs versus mt-CCs (n = 3 independent replicates per cell line). **b** Heatmap and **c** quantification of multiplexed analysis of NFκB canonical signaling in fl-CCs, mt-CCs and their matched CAFs. *n* = 3 biological repeats. Data are normalized to fl-cells. **d** Western Blot analysis of pIκBα (S32) and IκBα at baseline in cancer cells and in their matched CAFs. **e** ELISA quantification of TNFα in conditioned media generated by fl-CCs and mt-CCs. *n* = 3 biological repeats. **f** Heat map and **g** quantification of multiplexed analysis of NFκB canonical signaling in fl-e-CAFs upon treatment with TNFα and in untreated mt-e-CAFs. **h** Western Blot analyses of pIκBα (S32) and IκBα in fl-e-CAFs upon stimulation with TNFα. **i** RT-qPCR analysis of HSPG2 expression in fl-e-CAFs upon treatment with TNFα. **j** Schematic of paracrine signaling between mt-CCs and the surrounding fibroblasts to drive HSPG2 secretion in the tumor microenvironment. **k** Representative images of HSPG2 staining and quantification of optical density in stromal areas of pancreatic tumors isolated from KPflC mice (*n* = 6) and KPC mice (*n* = 9). Individual data points are presented with mean values and SEM. Scale bar: 200 µm. *p < 0.05, **p < 0.01, ***p < 0.001, ****p < 0.0001

We next performed microarray transcript expression profiling and Gene Set Enrichment Analysis (GSEA) of fl-CCs and mt-CCs in order to identify genes that could trigger HSPG2 expression in CAFs in a paracrine manner (Supplementary Fig. 7a, b, Supplementary data 2 and GEO accession number GSE123646). Among the genes that were differentially regulated between fl-CCs and mt-CCs, we found that expression of NFκB-signaling pathway components such as TNFα signaling were enhanced in mt-CCs compared to fl-CCs (Fig. 6a and Supplementary Fig. 7c, d)^64^. Considering that NFκB signaling can induce HSPG2 expression in a paracrine manner^65^, we next assessed whether increased NFκB signaling in mt-CCs could be a driver of increased production of HSPG2 by CAFs.

Increased activation of the NFκB pathway in mt-CCs compared to fl-CCs was confirmed using multiplexed analysis of canonical NFκB signaling^66^, and this was mirrored in their matched CAFs (Fig. 6b quantified in c). This aligns with the enhanced secretion of HSPG2 observed in mt-e-CAFs compared to fl-e-CAFs (Fig. 4a-c). Similarly, WB analyses of pIκαB(S32) in cancer cells and their matched CAFs (Fig. 6d) as well as ELISA analysis of TNFα secretion (Fig. 6e) confirmed that NFκB signalling is upregulated in mt-cells compared to fl-cells^67^. To functionally test the paracrine signaling between CCs and CAFs, we next stimulated fl-e-CAFs with TNFα and found that NFκB signaling was induced upon treatment, to a similar level as for mt-e-CAFs at baseline (Fig. 6f–h). Next, RT-qPCR analysis demonstrated that HSPG2 expression is increased in fl-e-CAFs upon treatment with TNFα (Fig. 6i).

Together, this data suggest that paracrine signaling between cancer cells and the surrounding fibroblasts in tumors with a GOF mutant p53 is partly mediated by enhanced NFκB signaling, and this can stimulate HSPG2 expression and deposition in the ECM (Fig. 6j).

Lastly, stromal deposition of HSPG2 in a cohort of KPflC and KPC pancreatic tumors was assessed via IHC staining. Quantification of HSPG2 staining in stromal areas revealed that HSPG2 deposition in the stroma is increased in KPC tumors compared to KPflC tumors (Fig. 6k and Supplementary Fig. 7e). This confirms that in the GEMM setting, HSPG2 deposition is enhanced in the stroma of native pancreatic tumors hosting cancer cells with a GOF mutant p53 compared to null p53.

### mt-e-CAFs reduce chemotherapy efficacy

**Fig. 7:**
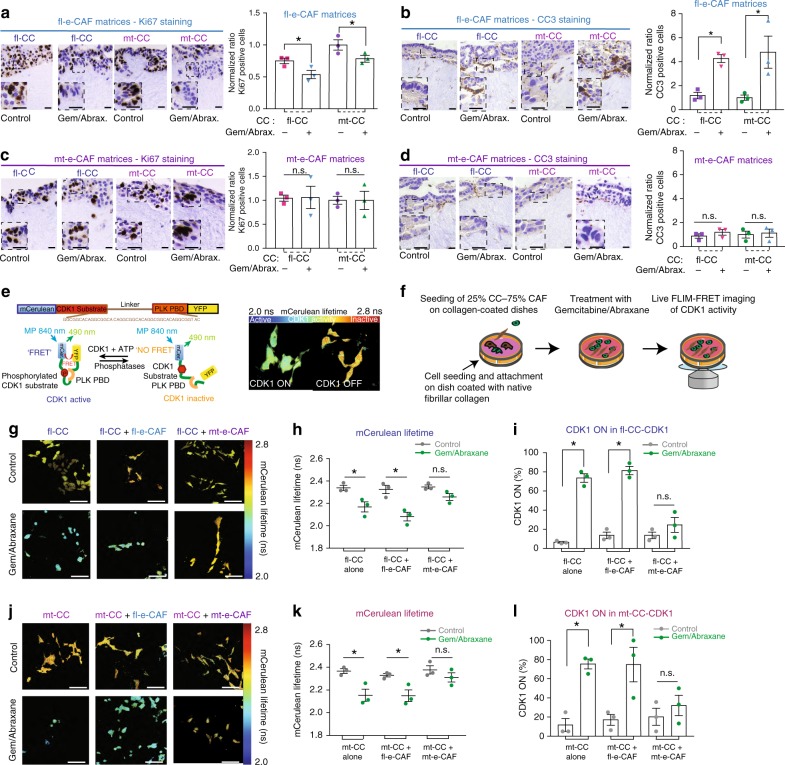
mt-e-CAFs impair cancer cell response to standard-of-care gemcitabine/Abraxane in vitro. **a** Representative images and quantification of Ki67 staining and of **b** cleaved-caspase 3 (CC3) staining of fl-CCs and mt-CCs invading into fl-e-CAF matrices upon treatment with control or with gemcitabine (100 nM)/Abraxane (100 nM) for 72 h. Scale bar: 50 μm, *n* = 3 biological repeats with three technical replicates per biological repeat. **c** Representative images and quantification of Ki67 staining and of **d** CC3 staining of fl-CCs and mt-CCs invading into mt-e-CAF matrices upon treatment with control or with gemcitabine (100 nM)/Abraxane (100 nM) for 72 h. Scale bar: 50 μm, *n* = 3 biological repeats with three technical replicates per biological repeat. **e** Schematic representation of the CDK1-FRET biosensor (adapted from 32) and representative mCerulean lifetime maps showing high CDK1 activity or low CDK1 activity as assessed via FLIM-FRET imaging of the CDK1-FRET biosensor. **f** Schematic representation of co-seeding of CAFs and cancer cells expressing the CDK1 biosensor on dishes coated with native fibrillar collagen, treatment with gemcitabine/Abraxane and FLIM-FRET imaging of CDK1 activity in cancer cells. **g** Representative mCerulean lifetime maps and **h** quantification of mCerulean lifetimes and **i**. CDK1 activity in fl-CCs cultured alone, cultured with fl-e-CAFs or with mt-e-CAFs and treated with control or with gemcitabine (100 nM)/Abraxane (100 nM) for 24 h. Scale bar: 100 μm, *n* = 3 biological repeats. **j** Representative mCerulean lifetime maps and **k** quantification of mCerulean lifetimes and **l** CDK1 activity in mt-CCs cultured alone, cultured with fl-e-CAFs or with mt-e-CAFs and treated with control or with gemcitabine (100 nM)/Abraxane (100 nM) for 24 h. Scale bar: 100 μm, *n* = 3 biological repeats, >100 cells analyzed per condition. Individual data points are presented with mean values and SEM. *p < 0.05

The role played by the tumor stroma^2^, particularly CAFs^28,32,68,69^ in tumor cell response to chemotherapy is becoming increasingly apparent. We therefore used IHC to assess the response of fl-CCs and mt-CCs to standard-of-care gemcitabine/Abraxane treatment during invasion into matrices containing fl-e-CAFs or mt-e-CAFs^22,60^. In fl-e-CAF remodeled matrices, gemcitabine/Abraxane reduced cell proliferation (Fig. 7a) and enhanced cell apoptosis (Fig. 7b) for both fl-CCs and mt-CCs, in line with the use of gemcitabine/Abraxane in the clinical setting^70^. Conversely in mt-e-CAF remodeled matrices, cell proliferation was not significantly decreased (Fig. 7c) and cell apoptosis was not significantly increased for either fl-CCs or mt-CCs (Fig. 7d) after treatment with gemcitabine/Abraxane compared to control. This implies that cancer cell response to chemotherapy can be modulated by the local CAF-generated microenvironment, or by changes in matrix stiffness^59^ (Fig. 1g and Supplementary Fig. 1d, e).

To assess whether direct contact between CAFs and cancer cells influenced response to chemotherapy, we seeded CCs and CAFs onto dishes coated with collagen and measured cell response to gemcitabine/Abraxane using IHC analyses (Supplementary Fig. 8). For fl-CCs seeded alone or in co-culture with fl-e-CAFs, cell proliferation was decreased and cell apoptosis was increased upon a 24 h exposure to gemcitabine/Abraxane (Supplementary Fig. 8a, b). However no significant changes of cell proliferation and cell apoptosis were observed in fl-CCs seeded with mt-e-CAFs and treated with gemcitabine/Abraxane for 24 h (Supplementary Fig. 8a, b). Similarly, cell proliferation was decreased and cell apoptosis was increased upon treatment with gemcitabine/Abraxane in mt-CCs seeded alone or with fl-e-CAFs. However, no significant change of cell proliferation and cell apoptosis were observed for mt-CCs seeded with mt-e-CAFs (Supplementary Fig. 8c, d).

Together, this demonstrates that mt-e-CAFs impair cancer cell response to gemcitabine/Abraxane (irrelevant of the cancer cell p53 status) by creating a protective environment but also via direct interactions with the cancer cells.

Although these IHC markers provide initial insights into cancer cell response to treatment, these fixed endpoints lack the ability to monitor single-cell behavior longitudinally in vivo. We recently employed a CDK1-FRET biosensor as a dynamic readout of cell response to gemcitabine/Abraxane with single-cell resolution in living mice (Fig. 7e^22,71–73^). The serine/threonine kinase Cyclin-dependent kinase 1 (CDK1) is a critical component of the complexes responsible for phosphorylating and activating target substrates controlling cell cycle progression. CDK1 is specifically activated in G2/M and monitoring its activity can be used as a surrogate indicator of cell cycle progression in response to gemcitabine/Abraxane^22,71,72^. In the fluorescence lifetime color map, high CDK1 activity (FRET) in response to gemcitabine/Abraxane treatment is shown by lower mCerulean lifetimes and blue/green colors, while low CDK1 activity (no FRET) is represented by longer mCerulean lifetimes and red/yellow colors (Fig. 7e).

We used the CDK1 biosensor as a readout of response to gemcitabine/Abraxane for fl-CCs and mt-CCs seeded with fl-e-CAFs or mt-e-CAFs on dishes coated with collagen. In agreement with earlier findings using IHC (Supplementary Fig. 8), CDK1 activity was increased after 24 h of treatment with gemcitabine/Abraxane for fl-CCs seeded alone or with fl-e-CAFs; however, there was no significant change of CDK1 activity for fl-CCs seeded with mt-e-CAFs and treated with gemcitabine/Abraxane for 24 h (Fig. 7g quantified in Fig. 7h, i). Similarly, while CDK1 activity was increased upon a 24 h treatment with gemcitabine/Abraxane in mt-CCs seeded alone or with fl-e-CAFs, there was no significant change of CDK1 activity in mt-CCs seeded with mt-e-CAFs and treated with gemcitabine/Abraxane for 24 h (Fig. 7j quantified in Fig. 7k, l).

**Fig. 8:**
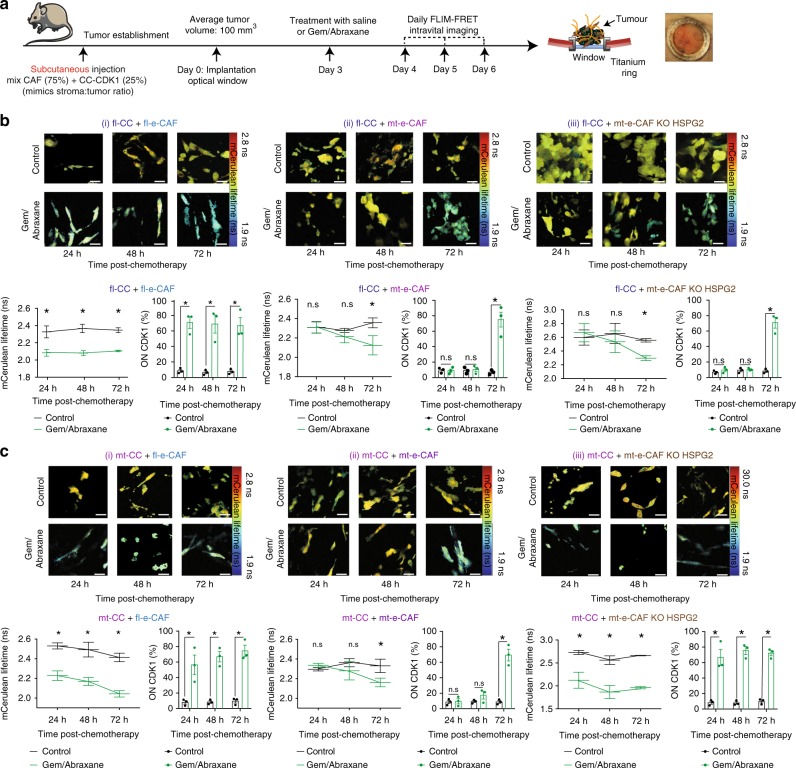
mt-e-CAFs delay fl-CC and mt-CC response to chemotherapy in an HSPG2-dependent manner for mt-CCs. **a** Schematic of timeline of subcutaneous injection of cancer cells with CAFs, tumor development, titanium window implantation, drug treatment, and longitudinal imaging. **b** Representative mCerulean lifetime maps (top images) and longitudinal quantification of mCerulean lifetimes (lower left hand panels) and of CDK1 activity (lower right hand panels) in fl-CCs injected with (i) fl-e-CAFs, with (ii) mt-e-CAFs WT or with (iii) mt-e-CAFs KO HSPG2 (mixed population) 24 h, 48 h, and 72 h after treatment with control or with gemcitabine/Abraxane. Scale bar: 100 μm, *n* = 3 mice per group. **c** Representative mCerulean lifetime maps (top images) and longitudinal quantification of mCerulean lifetimes (lower left hand panels) and CDK1 activity (lower right hand panels) in fl-CCs injected with (i) fl-e-CAFs, with (ii) mt-e-CAFs WT or with (iii) mt-e-CAFs KO HSPG2 (mixed population) at 24 h, 48 h, and 72 h after treatment with control or with gemcitabine/Abraxane. Scale bar: 100 μm, *n* = 3 mice per group. The threshold was calculated based on values between untreated and treated mice at 24 h post-treatment with gemcitabine/Abraxane for each group of cells co-injected subcutaneously. See Supplementary information for details on calculation of threshold. Individual data points are presented with mean values and SEM. *p < 0.05

We next used titanium optical windows to monitor cancer cell response to gemcitabine/Abraxane longitudinally in the presence of fl-e-CAFs or mt-e-CAFs within live tumors. As previously, CCs were co-injected with CAFs into the rear flank of Balb/c-Fox1nuAusb mice (Fig. 8a). Once tumors were fully established (average tumor volume: 100 mm^3^), optical titanium imaging windows were surgically implanted^74,75^, mice were administered with a single dose of gemcitabine/Abraxane on day 3 post-surgery (Fig. 8a) and imaging of CDK1 activity in cancer cells was performed over three days post-treatment (Fig. 8a). This revealed a sustained activation of CDK1 in fl-CCs at 24 h, 48 h, and 72 h after treatment with gemcitabine/Abraxane in tumors formed by fl-CCs co-injected with fl-e-CAFs (Fig. 8b (i)). This increase of CDK1 activity was only observed 72 h after treatment with gemcitabine/Abraxane in tumors formed by fl-CCs co-injected with mt-e-CAFs (Fig. 8b (ii)), revealing a delay of cancer cell response to chemotherapy in the presence of mt-e-CAFs in live tumors. Similarly, for tumors formed by mt-CCs injected with fl-e-CAFs, CDK1 activation was observed at 24 h, 48 h, and 72 h after treatment with gemcitabine/Abraxane (Fig. 8c (i)), however CDK1 activation only occurred after 72 h of treatment with gemcitabine/Abraxane in tumors generated by mt-CCs injected with mt-e-CAFs (Fig. 8c (ii)). Importantly, mt-e-CAFs KO HSPG2 did not significantly alter fl-CC response to gemcitabine/Abraxane over time compared to mt-e-CAFs WT (Fig. 8b (iii)). On the other hand, mt-CC response to gemcitabine/Abraxane occurred earlier when co-injected with mt-e-CAFs KO HSPG2 (CDK1 activation now at 24 h post-treatment, Fig. 8c (iii)).

**Fig. 9:**
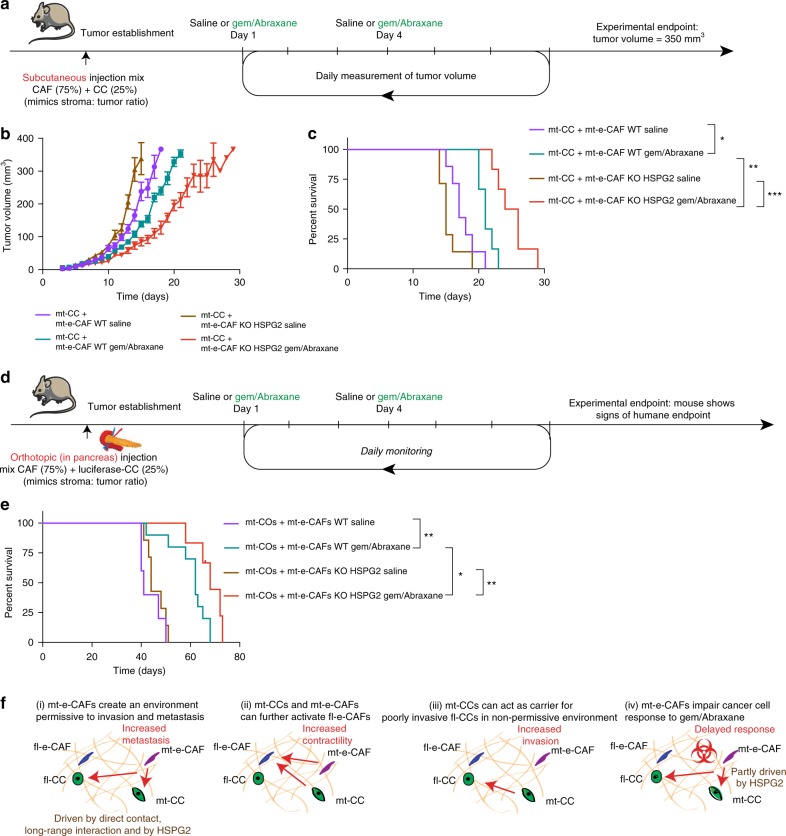
Loss of HSPG2 in the stroma improves the efficacy of gemcitabine/Abraxane in vivo. **a** Schematic representation of subcutaneous injection and treatment timeline, **b** quantification of tumor volume and **c**. time to experimental endpoint for mice subcutaneously injected with mt-CCs + mt-e-CAFs WT and treated with saline (*n* = 7) or with gemcitabine/Abraxane (*n* = 6) or with mt-CCs + mt-e-CAFs KO HSPG2 and treated with saline (*n* = 7) or with gemcitabine/Abraxane (*n* = 6). **d** Schematic representation of orthotopic (in pancreas) injection and treatment timeline, and **e** survival for mice orthotopically injected with mt-COs + mt-e-CAFs WT and treated with saline (*n* = 5) or with gemcitabine/Abraxane (*n* = 10) or with mt-CCs + mt-e-CAFs KO HSPG2 and treated with saline (*n* = 7) or with gemcitabine/Abraxane (*n* = 5). **f** Schematic summary of main findings. *p < 0.05, **p < 0.01, ***p < 0.001

This prompted us to assess whether long-term survival could also be improved in this setting. mt-CCs were subcutaneously injected with mt-e-CAFs WT or mt-e-CAFs KO HSPG2. Once tumors were established, mice were subjected to cycled administration of saline control or gemcitabine/Abraxane, until reaching experimental endpoint (Fig. 9a). Tumor growth was reduced and time to experimental endpoint (maximum tumor volume) was increased upon reduced deposition of stromal HSPG2 and in response to gemcitabine/Abraxane (Fig. 9b, c; compare gemcitabine/Abraxane in mt-CCs + mt-e-CAFs WT (green line) vs gemcitabine/Abraxane in mt-CCs + mt-e-CAFs KO (red line)). To further assess how reduction of HSPG2 in the stroma affects survival upon treatment with gemcitabine/Abraxane, we orthotopically injected mice with mt-CCs expressing luciferase in combination with either mt-e-CAFs WT or with mt-e-CAFs KO HSPG2. Once tumors were confirmed via IVIS imaging, mice were subjected to cycles of treatment with gemcitabine/Abraxane twice weekly (Fig. 9d). Similar to our findings using subcutaneous xenografts, we observed a prolonged time to experimental endpoint for mice injected with mt-e-CAFs KO HSPG2 (red) compared to mt-e-CAFs WT (green) upon treatment with gemcitabine/Abraxane (Fig. 9d, e). While in previous experiments we used IVIS imaging to determine experimental endpoint (Fig. 4h), here endpoint was reached when mice showed clinical signs of illness. This could explain the observed differences in time to experimental endpoints (Fig. 4h vs Fig. 9e). Together, our data demonstrate that reduced response to gemcitabine/Abraxane in the presence of mt-e-CAFs can be partly driven by HSPG2. This also suggests that targeting of stromal HSPG2 could potentially increase the efficacy of standard-of-care chemotherapy.

Collectively, this data demonstrate that interactions between cancer cells and mt-e-CAFs delay CC response to standard-of-care chemotherapy, and this is partly influenced by HSPG2 for mt-CCs but not for fl-CCs. This highlights a potential TP53 alteration-specific interaction between cancer cells and CAFs that governs response to chemotherapy.

## Discussion

PC development often relies on the establishment of a fibrotic stroma, which leads to aberrant interactions between cancer cells and stromal compartments^2^. Studies assessing stromal manipulation have highlighted how the pancreatic stroma, and in particular CAFs, play multiple, and often conflicting roles in driving disease progression^32,34,36,37^. While the molecular landscape of PC has been shown to be highly heterogeneous^5^, recent work has revealed the existence of distinct CAF populations with heterogeneous histologic, epigenetic, immunologic, and mechanical signatures within pancreatic tumors^30,37^.

Here, we generated CAFs from end-stage, poorly-metastatic KPflC and highly-metastatic KPC GEMMs of PC to interrogate how alterations in TP53, one of the most commonly altered genes in cancer, differently shape the pancreatic stroma. We reveal that CAFs educated by cancer cells with a GOF mutant p53 govern the establishment of an environment permissive to invasion and metastasis for GOF and null p53 cancer cells alike (Fig. 9f (i)).

We identified and validated HSPG2 as a critical component of the pro-metastatic environment created by mt-e-CAFs (Fig. 9f (i)). Our results indicate that reducing stromal deposition of HSPG2 impairs the ability of both fl-CCs and mt-CCs to invade into the surrounding matrix and spread to distant sites. The observed decrease of metastasis upon loss of stromal HSPG2 may potentially be due to disrupted cell adhesion or compromised integrin signaling, which can be regulated by HSPG2^26,51,65,76,77^. In addition, our data reveal that mt-CCs signal to the surrounding fibroblasts and induce HSPG2 expression in the stroma in a paracrine manner, which is partly mediated by NFκB signaling. This aligns with recent work in prostate cancer^65^, and suggests that targeting NFκB paracrine signaling could also be used in the future to disrupt stromal education driven by PC cells with a GOF mutant p53.

Interrogating the interactions between distinct cancer cells and CAFs demonstrated that fl-e-CAFs can be reprogrammed by interacting with mt-CCs or with mt-e-CAFs, leading to the acquisition of more invasive and metastatic features (Fig. 9f (ii)). In addition, using mosaic cultures of fl-CCs and mt-CCs, we found that invasive mt-CCs can act as a carrier for poorly invasive fl-CCs to increase their invasiveness in non-permissive environments (Fig. 9f (iii)). This is potentially mediated by the ability of mt-CCs to educate fl-e-CAFs to create a more permissive environment (Fig. 2b). It is also possible that the highly invasive mt-CCs create tracks in matrices generated by fl-e-CAFs, which can subsequently be followed by fl-CCs^56^. Our data suggest that within pancreatic tumors hosting heterogeneous subpopulations of cancer cells and CAFs, pro-invasive phenotypes can be transferred to less aggressive cells across the tumor to increase invasion and metastasis, and to impair response to chemotherapy.

Lastly, we revealed that mt-e-CAFs provide cancer cells with stromal cues to delay and reduce response to gemcitabine/Abraxane, which could be reverted by reducing HSPG2 stromal deposition for mt-CCs (Fig. 9f (iv)). This highlights a p53-dependent interaction between cancer cells and CAFs during response to gemcitabine/Abraxane. This also suggests that combining biomarkers of GOF mutant p53 in the cancer cell compartment with high HSPG2 stromal deposition may be used in the future to identify patients that could benefit from HSPG2 manipulation in combination with chemotherapy. Considering that HSPG2 secretion in the stroma is partly mediated by NFκB signaling, our findings warrant further consideration to repurposing anti-NFκB drugs that are currently used in the clinic^78–80^.

We propose that rather than completely inhibiting stroma-cancer cell feedback, specific manipulation of CAF subpopulations that have been educated by cancer cells harboring a GOF mutant p53 can impair metastasis while maintaining the anti-tumorigenic functions of the PC stroma.

## Methods

### Study design

Organotypic contraction and invasion assays as well as CDM−based experiments were performed in three independent biological repeats, with three technical replicates per repeat and per group. The numbers of mice used for each experiment are outlined in their corresponding figure legends. CDK1 activity was assessed via FLIM-FRET measurements in >40 cells per technical replicate or per mouse. IHC, SHG, picrosirius red, and GLCM analyses were conducted on three regions of interest in organotypic matrices and in five representative fields of view in subcutaneous xenograft and autochthonous experiments. Secretome analysis by mass spectrometry was performed on 4–5 biological replicates. Endpoints for animal experiments were defined according to Garvan Ethics Committee guidelines (16/13 protocol).

### Statistical analysis

Unpaired two-group comparisons were performed using the Mann–Whitney U test, unpaired multi-group comparisons by the Kruskal–Wallis test, and Kaplan–Meier curves were compared using the Mantel–Cox log-rank test. Where multiple tests were performed, familywise error rate was controlled by the Holm-Sidak step-up method. GraphPad Prism v7 was used for all procedures. Summary data in figures are presented as mean values with standard error of the mean (SEM), with asterisks used as a guide to statistical significance, as *p < 0.05, **p < 0.01, ***p < 0.001, ****p < 0.0001.

### Animals

Animal experiments were performed following the Australian code of practice for the care and use of animals for scientific purposes and in compliance with Garvan Ethics Committee guidelines (16/13 protocol), with genotyping of GEMMs performed by Garvan Molecular Genetics facility (Sydney, Australia).

### Establishment of cell lines and cell culture

Primary mt-CCs were isolated from *Pdx1-Cre, LSL-K-ras*^*G12D/+*^*, LSL-Trp53*^*R172H/+*^ tumors; primary fl-CCs were isolated from *Pdx1-Cre, LSL-K-ras*^*G12D/+*^*, LSL-Trp53*^*fl/+*^ tumors;^5,14^ primary mt-e-CAFs were isolated from *Pdx1-Cre, LSL-K-ras*^*G12D/+*^*, LSL-Trp53*^*R172H /+*^*; LSL-E-Cadherin-GFP* tumors; fl-e-CAFs were isolated from *Pdx1-Cre, LSL-K-ras*^*G12D/+*^*, LSL-Trp53*^*fl/+*^*; LSL-E-Cadherin-GFP* tumors. Also see ref. ^39^ for details on the generation of the LSL-E-Cadherin-GFP mouse. Isolation of cancer cells was previously described^18^. Cancer cells were maintained in Dulbecco’s modified Eagle media (DMEM) supplemented with 10% FBS and 1% penicillin/streptomycin in 20% O_2_ and 5% CO_2_. Cells were tested for the presence of mycoplasma (negative results), and their identity and purity throughout our in vitro experiment were confirmed via morphological analysis and growth properties. For isolation of CAFs, mice bearing well-established tumors (>150 days old) were sacrificed; primary tumors were surgically excised, mechanically dissociated in a tissue culture hood using a scapel blade and plated into tissue culture flasks in DMEM supplemented with FBS (10%) and 1% penicillin/streptomycin in 20% O_2_, 5% CO_2._ After one week of culture, cells had attached and grown on the tissue culture dish and primary CAF cultures were then enriched and purified using a FACS Aria III Cell Sorter (BD Biosciences, USA). Single-cell suspensions of 1 × 10^6^ cells were incubated with the anti-CD16/CD32 antibody (Mouse BD Fc Block; 1:200, BD Biosciences) in FACS buffer (PBS, 2% FBS, 2% HEPES) to block non-specific antibody binding. Single-cell suspensions were then pelleted and resuspended in FACS buffer containing the following cocktail of antibodies: anti-EpCAM-PerCP/Cy5.5 (1:500, BioLegend®, Clone: G8.8), anti-CD140a-APC (1:100; BioLegend®, Clone: APA5) and anti-Podoplanin-PE (GP38) (1:1,000, BioLegend®, Clone: 8.1.1) for 20 min on ice. Cells were then washed twice in FACS buffer before being resuspended in FACS buffer containing DAPI (1:1,000; Invitrogen) to discriminate live and dead cells. CAFs were isolated according to the following cell surface markers: CD140a^+^/GP38^+^/EpCAM^−^/DAPI^−^. Following CAF isolation, CAF were re-plated on a tissue culture dish and expanded. To prevent alterations of CAFs phenotype due to tissue culture, we utilized low passages of CAFs in vitro and for in vivo injection. CAF activation and purity were validated using live imaging of E-Cadherin-GFP as well as IHC and IF analyses. CAFs that had been isolated based on gp38 and CD140a positive expression and EpCAM negative expression, and that were also FAP^+^, ACTA^+^, GFP^−^, keratin 19^−^ and E-Cadherin^−^ were used for experiments. Telomerase-immortalised fibroblasts (TIFs), used for the generation of cell-derived matrices (Supplementary Fig. 2d-h), were generated as previously described^22^. mt-CCs, fl-CCs, mt-e-CAFs, fl-e-CAFs, and TIFs were cultured in DMEM (Gibco) complemented with 10% FBS and 1% penicillin/streptomycin in 20% O_2_, 5% CO_2_. Cancer cells were engineered to express the mCerulean-YFP CDK1-FRET biosensor designed by Gavet and Pines^71,72^ or GFP using a third-generation lentiviral packaging system followed by selection using blasticidin or via FACS on an Aria III Cell Sorter (BD Biosciences, USA). CAFs were engineered to express t-RFP using a retroviral packaging system followed by enrichment by FACS sorting (FACS Aria III Cell Sorter (BD Biosciences, USA)). Mutant p53^R175H^ and PCB6 + 1 control (empty vector, EV) vectors were a kind gift from Karen Vousden (BICR, Glasgow, UK).

### Immunoblotting

Cells were lysed in protein extraction buffer (50 mM HEPES, 1% Triton-X-100, 0.5% sodium deoxycholate, 0.1% SDS, 0.5 mM EDTA, 50 mM NaF, 10 mM Na_3_VO_4_, and protease cocktail inhibitor (Roche)). Lysates were separated by electrophoresis on a 10% acrylamide Bis-Tris gel or on a 4–12% gradient acrylamide Bis-Tris gel. Proteins were subsequently transferred onto a PVDF membrane (Immobilon-P, Millipore), blocked with 5% milk or with 1% BSA, incubated with primary antibodies (IκBα (1:1000), Cell signalling #9246 and pIκBα (S32, 1:1,000), Cell Signaling #9246) overnight at 4 °C, and probed with HRP-linked secondary antibodies (GE Healthcare, 1:5,000 diluted in 1% milk or in 1% BSA). Signal was detected using ECL reagent (Pierce) on a Fusion Fx7 ECL Imager. Signal was normalized to GAPDH or to actin expression. Raw data of immunoblotting can be found in the source data file.

### Organotypic assays

Organotypic assays were adapted from previously published protocols^22^.

*Contraction assay:* Rat-tail tendon collagen was extracted with 0.5 mM acetic acid to a concentration of 2.5 mg per ml. 2 × 10^5^ fl-CAF or mt-CAF were embedded in 2.5 mL of rat-tail collagen I. Once polymerized, fibroblast-collagen matrices were allowed to contract in DMEM containing 10% FBS and 1% penicillin/streptomycin for 12 days (or for 24 days in extended contraction assay, Supplementary Fig. 1a), with a media renewal at day 6. For treatment with conditioned media, 50% DMEM containing 10% FBS and 1% penicillin/streptomycin was added with 50% of conditioned media (CM), and this was renewed at day 6. For pharmacological removal of CAFs after matrix establishment, matrices were treated with puromycin (5 μg per ml) for 48 h, followed by 3 × 30 min washes in warm PBS.

*Invasion assay:* After contraction, 4 × 10^4^ cancer cells (fl-CC, mt-CC or a 50:50 mix of fl-CC:mt-CC) were seeded on the contracted matrix in DMEM containing 10% FBS and 1% penicillin/streptomycin and allowed to grow for 4 days. The matrix was then transferred to a metal grid, raised to an air-liquid interface and cancer cells were allowed to invade for 12 days. For experiments with CAFs seeded in the liquid phase during invasion, DMEM only or 1 × 10^6^ fl-e-CAFs or mt-e-CAFs were seeded in DMEM containing 10% FBS and 1% penicillin/streptomycin into a 6-cm dish 24 h prior to transferring the metal grid supporting the matrices in the dish containing the CAFs. DMEM and CAFs were renewed every 72 h during the 12-day invasion phase. After invasion, organotypic matrices were imaged or fixed in 10% formalin and processed for histochemistry analyses. Invasive index was measured in three representative areas per matrix and as per the formula:

$${{\mathrm{Invasive}}\,{\mathrm{index}} = \frac{{{\mathrm{Number}}\,{\mathrm{of}}\,{\mathrm{invading}}\,{\mathrm{cells}}}}{{\left( {{\mathrm{Number}}\,{\mathrm{of}}\,{\mathrm{invading}}\,{\mathrm{cells}} + {\mathrm{number}}\,{\mathrm{of}}\,{\mathrm{non}} - {\mathrm{invading}}\,{\mathrm{cells}}} \right)}} \times 100}$$

Clusters of cells invading together were defined as more than four cancer cells in close proximity and invading together into the CAF-collagen matrix.

For assessment of Ki67 and cleaved-caspase 3 (CC3) staining, the ratio was normalized to the value for mt-CCs treated with control. For each experiment, cells were counted in three fields of view per matrix, with three matrices per repeats and three biological replicates in total.

### Imaging and quantification of collagen birefringence

Paraffin-embedded samples were cut into 4 µm sections and stained with 0.1% picrosirius red (Polysciences) for fibrillar collagen according to manufacturer’s instructions. Polarized light imaging was performed on a Leica DM 4000 microscope and using an Olympus U-POT polarizer in combination with an Olympus U-ANT transmitted light analyzer fitted to the microscope. For assessment of picrosirius red staining in GEMMs tumors, representative polarized light images and quantification of total coverage of picrosirius red-stained tumors sections with six regions of interest (ROI) per tumor were analyzed blinded to the tumor genotype. In each tumor, areas of necrosis (where present) and tumor boundaries with normal tissue were excluded from analysis.

### Immunohistochemistry staining

Tissues fixed in 10% buffered neutral formalin and embedded in paraffin were cut as 4 μm sections. Sections were de-paraffinized using xylene and rehydrated in graded ethanol washes. Antigen retrieval was achieved via target retrieval solution low-pH (DAKO S1699) for 30 min at 93 °C for organotypic samples and at 100 °C for mouse or human tissues. Slides were subsequently quenched in 3% H_2_O_2_ before application of protein block (Dako) and incubation with primary antibodies (ACTA 1:200, Abcam #ab5694; Pab421 1:100, Merck Millipore; cleaved-caspase 3 1:200, Cell Signalling #9661; Ki67 1:500 Thermo fisher; and A7L6 HSPG2 1:300, Merck Millipore). Secondary antibodies (Envision) coupled to HRP were then applied and detection was performed with diaminobenzidine (DAB). H&E staining and counterstaining were done on the Leica autostainer XL. Quantification of DAB intensity was performed in ImageJ. DAB optical density was measured using color deconvolution and the average DAB intensity was computed for each cell.

### Immunocytochemistry staining

Cells seeded on a glass coverslip were washed three times in warm PBS prior to fixation in 4% paraformaldehyde (PFA, 10 min, room temperature). Cells were subsequently permeabilized with 0.5% Triton-X-100 (10 min, room temperature), quenched with 3%H_2_O_2_ (30 min, room temperature), blocked with a protein block (Dako, 30 min, room temperature) and incubated overnight with primary antibodies (ACTA2 1:200, Abcam #ab5694; cleaved-caspase 3 1:200, Cell Signalling #9661; Ki67 1:500, Thermo Fisher, A7L6 HSPG2 1:300 (mAb targeting domain IV of mouse HSPG2, Merck Millipore; CCN1 1:1000 (anti-HSPG2 antibody, developed by the Whitelock laboratory) and t-RFP 1:100, Evrogen #AB233). Subsequently, secondary antibodies (Envision) coupled to HRP were applied and detection was performed with DAB.

### Immunofluorescence staining

Cells were seeded on glass coverslips, washed in PBS and fixed in 4% PFA (10 min, room temperature). Cells were permeabilzed using Triton-X-100 (10 min, room temperature), blocked with 2% BSA (2 h, room temperature) and incubated with primary antibodies (E-Cadherin 1:200, BD Biosciences #610182; FAP 1:500 Abcam #53066; keratin 19 1:50, DSHB USA) overnight at 4 °C. Cells were incubated with Alexa488- or AlexaCy5-coupled secondary antibody (Jackon ImmunoResearch Laboratories Inc., 1:5,000, 1 h at room temperature), counter-stained with DAPI, and mounted in Mowiol mounting medium.

### Second Harmonic Generation (SHG) imaging and analysis

Second Harmonic Generation imaging is a label-free imaging technique that allows imaging of non-centrosymmetric entities such as collagen fibers. Production of a SHG signal occurs when two photons with the same wavelength fuse into a single photon with half of the original wavelength upon interacting with a non-centrosymmetric entity. SHG signal was acquired using a Leica DMI 6000 SP8 inverted confocal microscope with a 25 × 0.95 NA water objective. The excitation source was a Ti:Sapphire femtosecond laser cavity (Coherent Chameleon Ultra II) operating at 80 MHz and tuned at a wavelength of 880 nm. Intensity was recorded with a RLD HyD detector (440/20 nm). Three 512 × 512 representative regions of interest were imaged per sample, with a line average of 3 and over a 3D z-stack (50 μm depth with a z-step size of 2.5 μm on fresh samples and 20 μm depth with a z-step size of 1.52 μm on cut tissue sections). SHG signal intensity was measured using Matlab (MathWorks). Representative images of maximum intensity projections are shown.

### Grey-level co-occurrence matrix

Collagen fiber network organization was characterized using grey-level co-occurrence matrix (GLCM) analysis. This method provides a readout of the texture of a sample by quantifying the similarity between pixels across an image^44^. The correlation curves represent the similarity in signal intensity between pixels acquired via single-plane SHG imaging of collagen fibers (line average for SHG acquisition: 16). A slower decay shows a more organized network of collagen fibers than in samples with a faster decay. GLCM analysis was performed in Matlab (Mathworks) as previously described^44^. The GLCM correlation parameter for each image was calculated using looped operation of the plug-in and for 0°, 90°, 180°, and 270° directions. Normalized correlations were calculated in MATLAB (MathWorks) and the mean correlation was plotted against the neighbor index in GraphPad software.

### Shear rheology measurements

Storage modulus in matrices remodeled by CAFs was measured on a Discovery Series Hybrid DHR-3 controlled stain rotational rheometer (TA Instruments) using a 8 mm sandblasted parallel plate geometry. Following calibration of the rheometer according to the manufacturer’s instructions, the axial force was set to zero and the CAF matrices were subjected to a controlled strain with a continuous oscillation and with an oscillation frequency of 0.5 rad per sec; an oscillation strain ranging from 0.2% to 2.0% and an axial force of 0.03 N; a conditioning time of 2.0 s and a sampling time of 3.0 s. Ten data points per decade were acquired. Storage modulus was determined as the mean value within the linear viscoelastic range.

### Atomic force microscopy (AFM) measurement of matrix stiffness

CAF-collagen matrices were immobilized on a 40 mm glass bottom cell culture dish using a 10% agarose solution (Bioline Australia). Nano-indentation was performed on a Bioscope Catalyst (Bruker) mounted on a TMC anti-vibration table (Technical Manufacturing Corporation) and using a 1 μm spherical colloidal probe (Novascan) with a spring constant of 0.06 N per m mounted on the fluid holder of the AFM scanner. Prior to measurements, AFM calibration was performed by measuring the deflection sensitivity of the probe in fluid by engaging the probe on an uncoated glass substrate, withdrawal of the probe, and then using a thermal tune sweep to determine the spring constant. Indentation was performed using a peak force tapping mode (average loading force of 1 nN) on three different areas per matrix and ten indentations points per area. The Hertz spherical indentation model was used to calculate the Young’s modulus values^22^.

### Media conditioning

Cancer cells and CAFs plated in a tissue culture dish and reaching 75% confluence were washed three times using phosphate-buffered saline and were subsequently cultured for 24 h in DMEM Phenol Red free (Gibco) media complemented with 1% penicillin/streptomycin in 20% O_2_/5% CO_2_. Conditioned media were then collected, passed through a 0.2 μm filter and spun at 200 g for 10 min to remove cell debris. Conditioned media were either used fresh or stored at −80 ^o^C before being used during contraction assays. For immunoblotting and mass-spectrometry assessments, conditioned media were concentrated using ultrafiltration columns (3-50 kDa →100 kDa, Sartorius) and normalized to a concentration of 20 μg per 10 μl.

### Real-time quantitative polymerase chain reaction

RNA samples were isolated at the Garvan Molecular Genetics facility using the Macherey–Nagel Nucleospin RNA plus kit, according to the manufacturer’s instructions. RNA was reverse transcribed with the Transcriptor First Strand cDNA Synthesis Kit (Roche Diagnostics) and cDNA was synthesized from 1 μg of total RNA and diluted 1:10. Subsequent RT-qPCR experiments were performed using the Roche Universal Probe Library System on a Roche LightCycler480® (Roche LifeScience). Probes and programs used for RT-qPCR analysis are described in Supplementary Data 3 and 4.

Relative mRNA expression levels were normalized to *GAPDH* and quantification was performed using the comparative CT method described previously^43^ for each biological replicate.

### CDM establishment and monitoring of cell streaming

CDMs were generated as previously described^54^. In brief, confluent Telomeraze-immortalized fibroblasts were exposed to 50 μg per ml ascorbic acid for 7 days prior to denuding cells. For streaming experiment, cells (25% GFP-cancer cells and 75% RFP-CAFs) were seeded onto the resulting CDMs and longitudinally imaged using an Incucyte over 72 h. Cell streaming anisotropy was quantified using FibrilTool^55^ in the GFP channel to measure directionality-dependent coordinated cell migration, as previously performed^22^.

### Local invasion in subcutaneous xenograft experiment

1×10^6^ cells (25% cancer cells, 75% CAFs) were resuspended as single cells in 50 μl PBS, maintained on ice before being subcutaneously injected into the rear flank of BALB/c-Fox1nuAusb mice under anaesthesia (isoflurane 3 L, O_2_ 1 L min per, vacuum was used constantly to remove excess of O_2_). Tumor volume was measured daily. When average tumor volume reached 100 mm^3^, mice were sacrificed and subcutaneous xenografts were collected and fixed in 10% formalin buffer. Cell invasion into subcutaneous fat, muscle, and skin was analyzed using the scoring system described in Supplementary Fig. 3. This was scored blinded by two separate researchers, as previously described^25^.

### Orthotopic injections and monitoring of tumor progression

Cancer cells and CAFs were resuspended as single cells, counted on an automated cell counter, serially diluted to obtain a mix of 50 luciferase-cancer cells and 150 CAFs. Cells were mixed in a volume of 50 μL PBS/Matrigel (1:1 mix), maintained on ice before being injected into the pancreas of NOD/SCID/ILR2γ mice under anesthesia (isoflurane 3 L, O_2_ 1 L per min, vacuum was used constantly to remove excess of O_2_) during open laparotomy. Tumor growth and metastatic spread were monitored twice weekly via IVIS imaging. Mice were administered with Luciferin (150 mg per kg, Gold Biotechnology) via intraperitoneal injection and signal was acquired on an IVIS Spectrum with open filters and small binning. Time to metastasis was determined when Luciferase signal was detected in areas outside the pancreatic zone. Experimental endpoints were defined as development of general signs of disease/discomfort (dehydratation, prolonged hunching, ruffled coat, fluid built up in the abdomen, abdominal distension, reduced movement/reactivity, obvious lesions or huddling in cage corner) or weight loss ≥10% of body weight.

In this manuscript, we utilized both subcutaneous xenografts and orthotopic injections of pancreatic cancer cells with CAFs. The subcutaneous model is a rapid model, which we and others previously optimized and employed for rapid assessment of early tumor growth, local invasion and response to treatments^22,25^. The orthotopic model was optimized to monitor distinct steps of tumor progression, onset of metastasis and response to treatment in a native tissue.

In the subcutaneous xenograft experiment presented in Fig. 9, experimental endpoint was determined as maximum tumor volume of 350 mm^3^ (measured with calipers). In the orthotopic model, determination of tumor burden and experimental endpoint in Fig. 4 was achieved via IVIS imaging. However, in Fig. 9, the additional burden of administration of gemcitabine/Abraxane precluded IVIS imaging and experimental endpoints were determined using clinical signs of illness (including but not limited to change in animal behavior, prolonged hunching, development of ascites, apathy, overnight weight loss >10%).

Determination of the number of mice required to detect statistical significance was performed in line with 3Rs requirements. For changes in local invasion, we estimated an incidence of local invasion in ~75% of mice injected with mt-CCs (based on previously published work^22,25,39^). Changes in the incidence of metastasis to ~25% was expected, and the predicted standard deviation was 20%. Using these figures, and type-1 error α = 0.05, we could identify a difference in local invasion to a power of β = 80% at a significance level of 5% in cohorts of four mice per group. Statistical assessments were performed using unpaired multi-group comparisons by the Kruskal–Wallis test. However, to account for censored events, where tumor growth may not occur, an additional mouse was included per group.

### Microarray transcript expression profiling

Total RNA was isolated using the RNeasy extraction kit (Qiagen) and measured on the 2100 Bioanalyzer (Agilent). Total RNA was converted to cDNA, biotin labeled and then hybridized to the Affymetrix mouse GeneChip 430 2.0 Arrays, as per the manufacturer’s instructions (Affymetrix Ca, USA), at the Paterson/Beatson Institute (UK). All cells were prepared in biological triplicate for each experimental group. Quality control was performed using the affy R package^81^. Normalization and probe-set summarization was performed using the robust multichip average (RMA) method^82^. Differential expression between experimental groups was performed using Partek® Genomics Suite® software, Copyright^©^; Partek Inc., St. Louis, MO, USA. All transcript probe-sets with an adjusted step-up *p*-value ≤ 0.05 were classed as differentially regulated between the samples. Where applicable, genes annotated to multiple probe-sets were collapsed to a single representative probe-set by selecting the one with the most significant adjusted *p*-value. Microarray data are freely available from GEO GSE123646.

A core set of genes (Bcl2, Cxcl1, Cxcl12, Nfkbia, Tnfrsf9, Tmfsf11) from the NFKB pathway were identified as significantly differentially regulated (adjusted *p*-value ≤ 0.05) between the Trp53 and Trp53 (R172H) cells. We then extended this to look at other members of the pathway, identifying a further 35 genes that showed significantly higher expression in the mt-CCs (mutant cancer cells Trp53 R172H) cells compared to the fl-CCs (Trp 53 knockout controls).

### GSEA analysis

Functionally associated gene-sets were identified using pre-ranked Gene Set Enrichment Analysis (GSEA) against the Hallmark (v6.2) gene-sets. For this the ranked gene list was derived from limma moderated t-statistics comparing the mt-CCs to the fl-CCs. Genes annotated to multiple probe-sets were collapsed to a single representative “best” probe-set by selecting the one with the largest absolute t-statistic. Mouse gene-symbols were mapped to their human orthologs using the ensembl database (version 94). Mouse genes with no annotated human ortholog were removed and mouse genes mapping to the same human gene ortholog were again collapsed using the “best” t-statistic approach.

### QuPath-based quantification of DAB staining in GEMMs

Pancreatic tumors from KPC and KPflC mice were stained for HSPG2 (CCN1 1:1000) and scanned using an Aperio slide scanner. In QuPath, all cells were detected and a classifier was built via manual detection of >100,000 cancer cells and stromal cells. The classifier was constructed using all detection parameters but excluding criteria based on DAB intensity. Next, the classifier was applied and we manually verified that detection of cancer cells and stromal area was accurate. QuPath was then employed to measure optical density in stromal areas.

### Proteomics analysis of CAF-derived conditioned media

Secreted proteins contained in conditioned media were lysed using denaturing lysis buffer containing 6 M urea, 2 M thiourea, and 100 mM HEPES (pH8). Protein concentration was measured using QuBit and samples were normalized to 1 μg per μl prior to reduction using 10 mM TCEP and alkylation with 40 mM chloroacetamide at room temperature for 30 min. Proteins were then pre-digested with LysC using 1:50 (enzyme:protein ratio), diluted 1:5 with 100 mM HEPES followed by an overnight digestion with trypsin using a 1:50 (enzyme:protein ratio) at 37 °C. The digests were acidified to a final concentration of 1% trifluoroacetic acid (TFA) and desalted using SDB-RPS stage tips. Columns were conditioned with 100% acetonitrile followed by 30% methanol containing 0.2% TFA and then re-equilibrated with 0.2% TFA. Samples were subsequently loaded on columns and washed with 0.2% TFA followed by 90% isopropanol containing 1% TFA and then eluted with 80% acetonitrile containing 5% ammonium hydroxide and dried by vacuum centrifugation.

Peptides were resuspended in 2% acetonitrile, 0.1% TFA and loaded onto a 50 cm × 75 µm inner diameter column packed in-house with 1.9 µm C18AQ particles (Dr Maisch GmbH HPLC) using an Easy nLC-1000 UHPLC operated in single-column mode. Peptides were separated using a linear gradient of 5–30% Buffer B over 150 min at 300 nL per min (Buffer A = 0.1% formic acid; Buffer B = 80% acetonitrile, 0.1% formic acid). The column was maintained at 60 °C using a PRSO-V1 ion-source (Sonation) coupled directly to a Q-Exactive mass spectrometer (MS). A full-scan MS1 was measured at 35,000 resolution at 200 m per z (300–1650 m per z; 100 ms injection time; 3e6 AGC target) followed by isolation of up to 15 most abundant precursor ions for MS/MS (2 m per z isolation; 27 normalized collision energy; 17,500 resolution at 200 m per z; 60 ms injection time; 1e5 AGC target). Raw data were processed using MaxQuant version 1.5.8.3 [PMID: 19029910] using all default parameters with MaxLFQ and match between runs enabled. Data were searched against the mouse UniProt database and filtered to a 1% false discovery rate (FDR) at the peptide spectral match and protein level. Data were further processed with Perseus version 1.5.3.0 [PMID: 27348712] using t-tests and ANOVA and adjusted for multiple hypotheses testing using permutation-based FDR to obtain q-values.

The volcano plot (Fig. 4a) represents the Student *t*-test statistic and the fold change between each condition for secreted proteins identified by proteomics analyses. The heat-map (Supplementary Fig. 5a) plots the absolute (log_2_) expression levels of each of the secreted proteins that are significantly differently expressed between fl-e-CAFs and mt-e-CAFs (also see Supplementary Data 1).

### Generation of KO and KRAB lines with CRISPR-Cas9

Mammalian codon optimized *S.Py*. Cas9 with C-terminal nucleoplasmin NLS tag (kind gift from Doudna lab) was cloned into a third-generation lentiviral vector downstream of an EFS promoter and fused to puromycin resistance protein with a P2A peptide. Single-guide RNA (*HSPG2* DNA target sequence (PAM): 5’-GCAGGTCCTCATCATCAGAG**(AGG)**-3’ located on exon 2 of mouse *HSPG2*) was also cloned into the same vector downstream of a U6 promoter. The Cas9 lentiviral vector was transfected into HEK293T cells together with psPAX and pMD2.G packaging and envelope vectors (gifts from Trono lab, Addgene #12260 and #12259 respectively) with PEI (Sigma). Lentiviral particles were harvested from culture media, filtered through 0.45 µm filter units and used to infect CAFs. After 48 h, CAFs were selected for puromycin resistance (40 µg per ml) and sorted by flow cytometry to obtain monoclonal populations. Editing was verified by Sanger sequencing at the genomic locus as follows: cells were lysed in QE buffer (Epicenter) at 65 °C for 20 min followed by 95 °C for 20 min; the locus amplified by PCR using Kapa HotStart polymerase (Fwd primer: 5’-CCTGCTCATGTCTTTCCAGGT-3’ and Rev primer: 5’-GCCTGGAACTCCTGATCCTT-3’) and sequenced. T7E1 assay was performed using the same primers provided in Supplementary Data 3.

For CRISPR-Cas9 interference (CRISPRi), *S.Py*. Cas9 was rendered catalytically inactive by introducing D10A and H840A mutations and cloned in frame with a KRAB domain at its N-terminus into the third-generation lentiviral vector. Single-guide RNA was designed to bind the following genomic DNA sequence (PAM): 5’- GAGCACGTGGTGTGAAGGAG(**CGG)**-3’ in the CpG islands upstream of *HSPG2* gene. Lentiviral particles were generated as described previously in HEK293T cells and used to infect CAFs. After puromycin selection, HSPG2 expression levels were assessed by RT-qPCR using probes targeting exons 4, 6–7, 22 and 74 to confirm repressed *HSPG2* expression.

### In vitro doubling time quantification

CAFs were pre-treated with mitomycin-c (10 μg per ml, 24 h) and were seeded with cancer cells (75% CAFs, 25% cancer cells). After 48 h of co-culture, cells were harvested and counted on a hematocytometer. Doubling time was calculated as per the formula: $DT = T \times \frac{{{\mathrm{ln}}2}}{{{\mathrm{ln}}\left( {\frac{{Xe}}{{Xb}}} \right)}}$, (with DT: doubling time, T: time of co-culture (48 h), Xe: cancer cell number at endpoint, Xb: cancer cell number at T = 0).

### ELISA

Quantification of TNFα in conditioned media was performed using ELISA kits (TNFα: Abcam #ab100747) as per the manufacturer’s instructions.

### Magpix analysis of canonical NFkB signalling

Multiplexed analysis on the Magpix platform was performed using a MILLIPLEXMAP NFkB Signaling Magnetic Bead Kit (#48-630MAG) and according to the manufacturer’s instructions.

### Implantation of titanium window for longitudinal imaging

Twenty-four hour prior to the surgery and for 72 h following implantation of the window, mice were kept on Carprofen (5 mg per kg, Rimadyl). Pain management was achieved via injection of buprenorphine (0.2 mg per kg, Temgesic) prior to surgery as well as 6 h post-surgery. Mice were anesthetized (isoflurane 3 L, O_2_ 1 L per min, vacuum was used constantly to remove excess of O_2_), the incision site was disinfected with 70% ethanol and an incision was made in the skin above the palpable subcutaneous xenograft. A purse string suture (Mersilk) was placed through the skin alone to create a continuous suture around the incision site. A titanium window ring with a 12-mm glass coverslip was then inserted into the incision site and the suture was tightened. Four days following surgery, mice were treated with saline or with gemcitabine/Abraxane and mice were imaged on day 5, 6 and 7 post-surgery.

### Drug treatment

Abraxane (Specialized Therapeutics) was administered to mice by intraperitoneal injection at 30 mg per kg and was used at 100 nM for in vitro experiments. Gemcitabine (Jomar Life Research) was administered to mice by intraperitoneal injection at 70 mg per kg and was employed at 100 nM for in vitro experiments. Recombinant mouse TNFα protein (Abcam #ab9740) was used in vitro at 10 ng per μl.

### FLIM-FRET imaging and analysis

FLIM-FRET imaging was performed using the system described above (SHG imaging). mCerulean was excited at 840 nm, and the signal was detected using a 483/32 nm filter. FLIM data were recorded using a Picoharp 300 TCSPC system (Picoquant). Images of 512 × 512 pixels were acquired with a line rate of 600 Hz. The pixel dwell time was 5 μs, and images were integrated until 500 photons per pixel were acquired. For in vivo measurements of mCerulean lifetimes in live xenografts, cancer cells expressing the CDK1-FRET biosensor were trypsinized and resuspended in cold phosphate-buffered saline (PBS) with CAFs. 1 × 10^6^ cells were injected subcutaneously into the rear flank of BALB/c-Fox1nuAusb mice under anesthesia (isoflurane 3 L, O_2_ 1 L per min, vacuum was used constantly to remove excess of O_2_). During imaging, the mouse was maintained under anesthesia (isoflurane 3 L, O_2_ 1 L per min) and placed on a 37 °C stage. mCerulean lifetimes were analysed as previously described^22^, by drawing regions of interest around subcellular areas and recording the lifetime (*τ*) of the single exponential function fit to the fluorescence decay data. Lifetime maps were generated with intensity thresholds set to the average background pixel value for each recording. The raw data were smoothened, and a standard rainbow color look up table (LUT) was applied, with lifetimes of 1.9–2.8 ns for mCerulean. Areas where no lifetime measurement above the background noise could be achieved are shown in black in the lifetime map.

In the control (untreated situation), the mCerulean lifetime is high while upon treatment with gemcitabine/Abraxane, the mCerulean lifetime decreases. Stratified readouts from the mCerulean lifetimes were used as a threshold for cells being classified as CDK1 high versus CDK1 low. The threshold lifetime was set as the midpoint between the population means of the untreated and treated groups. The same threshold was employed to classify cells in the in vitro experiments. For the longitudinal intravital imaging experiments, the threshold was calculated based on values between untreated and treated mice at 24 h post-treatment with gemcitabine/Abraxane for each group of cells co-injected subcutaneously. Single-cell FLIM-FRET imaging of 60 cells per tumor in 5 different areas of the tumor and in three mice per treatment group were imaged.

### Assessment of mouse survival in the subcutaneous tumor model

1 × 10^6^ cells (25% cancer cells, 75% CAFs) in 50 μl PBS were subcutaneously injected into the rear flank of BALB/c-Fox1nuAusb mice under anaesthesia (isoflurane 3 L, O_2_ 1 L per min, vacuum was used constantly to remove excess of O_2_). Once mice developed palpable tumors, mice treatment with saline control or with gemcitabine/Abraxane was initiated. Mice were treated twice weekly, on day 1 and day 4. Tumor volume was measured daily. Experimental endpoint was reached when tumor volume reached 350 mm^3^.

### Assessment of mouse survival in the orthotopic model

Fifty cancer cells expressing luciferase and 150 CAFs in 50 μl PBS:Matrigel (1:1) mix were injected into the pancreas of NOD/SCID/ILR2γ mice under anesthesia (isoflurane 3 L, O_2_ 1 L per min, vacuum was used constantly to remove excess of O_2_) during open laparotomy. Once tumor establishment was confirmed via IVIS whole-body imaging, mice entered cycles of treatment with gemcitabine and Abraxane twice weekly. Mice were monitored daily, and when mice showed signs of clinical illness (dehydratation, prolonged hunching, ruffled coat, fluid built up in the abdomen, abdominal distension, reduced movement/reactivity, obvious lesions, or huddling in cage corner) or weight loss ≥10% of body weight, they were sacrificed and tissues were collected for further analysis.

### Reporting summary

A reporting summary for this article is available as a Supplementary Information file.

## Supplementary information

Supplementary Information

Description of Additional Supplementary Files

Supplementary Data 1

Supplementary data 2

Supplementary data 3

Supplementary data 4

Reporting summary

Source Data

## Data Availability

The microarray data have been deposited in the GEO database under the accession code GSE123646. The source data underlying Fig. 1-9 are provided as a Source Data file. All the other data supporting the findings of this study are available within the article and its supplementary information files and from the corresponding author upon reasonable request.

## Code availability

GLCM analysis was performed using previously published code^8^, the code for FLIM analysis was developed by Dr. Aleš Benda and as previously published^8^.
