# Supplementary material for: CAF hierarchy driven by pancreatic cancer cell p53-status creates a pro-metastatic and chemoresistant environment via perlecan
Source: Nat Commun. 2019 Aug 12;10:3637. doi: 10.1038/s41467-019-10968-6 (PMC6691013; doi:10.1038/s41467-019-10968-6)
Supplement: Supplementary file 1 — Supplementary Information [file 41467_2019_10968_MOESM1_ESM.pdf]

CAF hierarchy driven by pancreatic cancer cell p53-status creates a pro-metastatic and chemoresistant environment via perlecan.

Vennin *et al.*

Supplementary figure 1

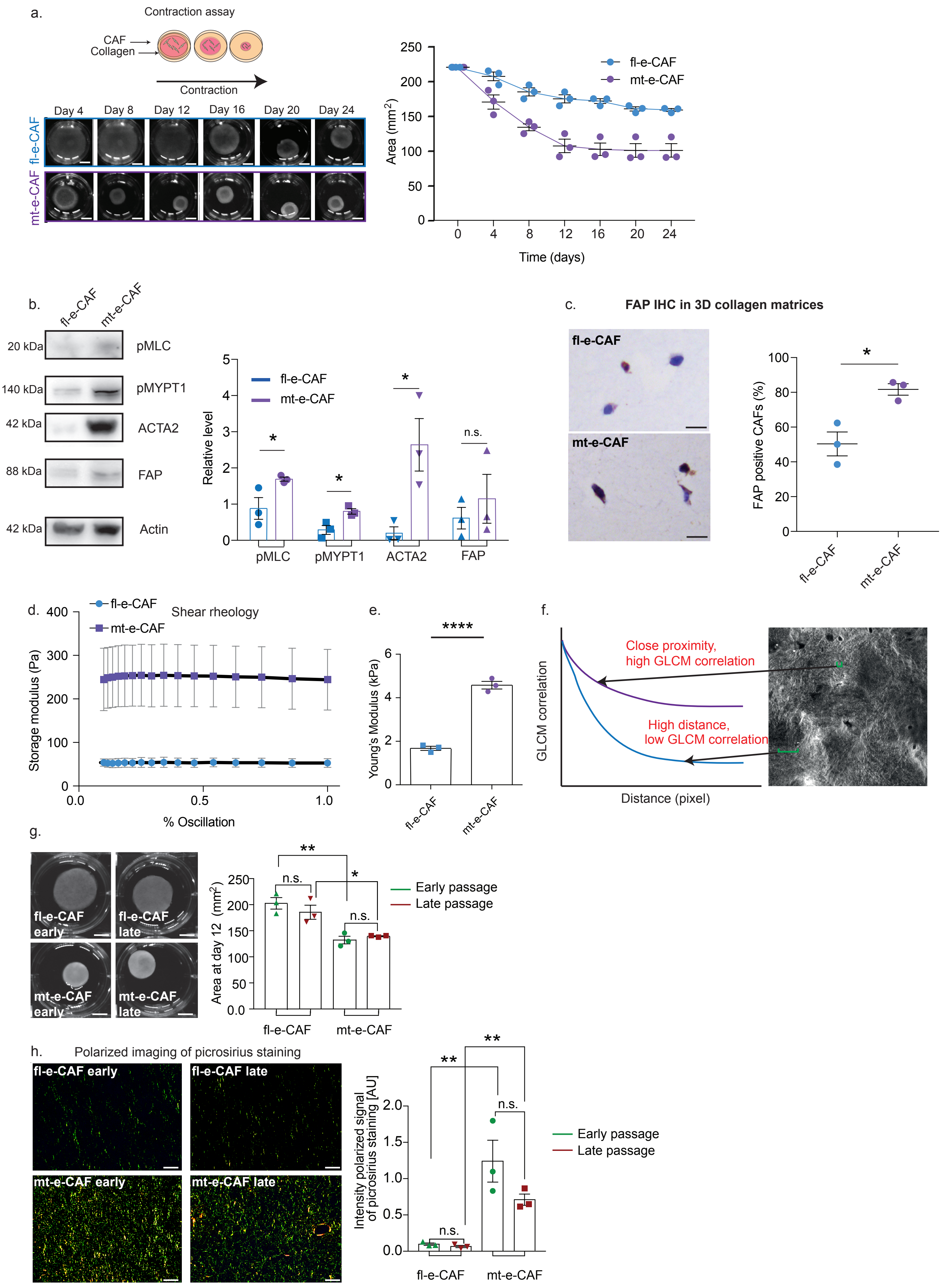

Supplementary figure 1. ECM remodeling is increased in mt-e-CAFs compared to fl-e-CAFs.

a. Schematic representation of CAF-driven contraction assay, representative images of CAF-collagen matrices and quantification of matrix area in matrices remodeled by fl-e-CAFs or by mt-e-CAFs during an extended contraction assay (24 days). Scale bar: 1 cm, n=3 biological repeats with three technical replicates per biological repeat. b. Immunoblotting analysis of markers of CAF contractility and CAF activation in fl-e-CAFs and mt-e-CAFs. n=3 biological repeats. c. Representative images and quantification of FAP positive cells in collagen matrices remodeled by fl-e-CAFs or by mt-e-CAFs. Scale bar: 100 µm, n=3 biological repeats with three technical replicates per biological repeat. d. Quantification of storage modulus as a function of % oscillation using shear rheological assessment in matrices remodeled by fl-e-CAFs or by mt-e-CAFs. n=3 biological repeats with three technical replicates per biological repeat. e. Atomic force microscopy quantification of the Young's modulus of matrices generated by fl-e-CAFs and by mt-e-CAFs. f. Schematic representation of GLCM analysis of the texture of collagen fiber network in CAF-collagen matrices. g. Representative images of matrices and quantification of matrix area after a 12-day contraction assay using fl-e-CAFs and mt-e-CAFs at early and late passages. n=3 biological repeats with three technical replicates per biological repeat, scale bar: 1cm. h. Representative images and quantification of polarized imaging of picrosirius staining in matrices established by fl-e-CAFs and mt-e-CAFs at early and late passages. n=3 biological repeats with three technical replicates per biological repeat, scale bar 50 µm. Individual data points are presented with mean values and SEM. * $p < 0.05$, ** $p < 0.01$.

Supplementary figure 2

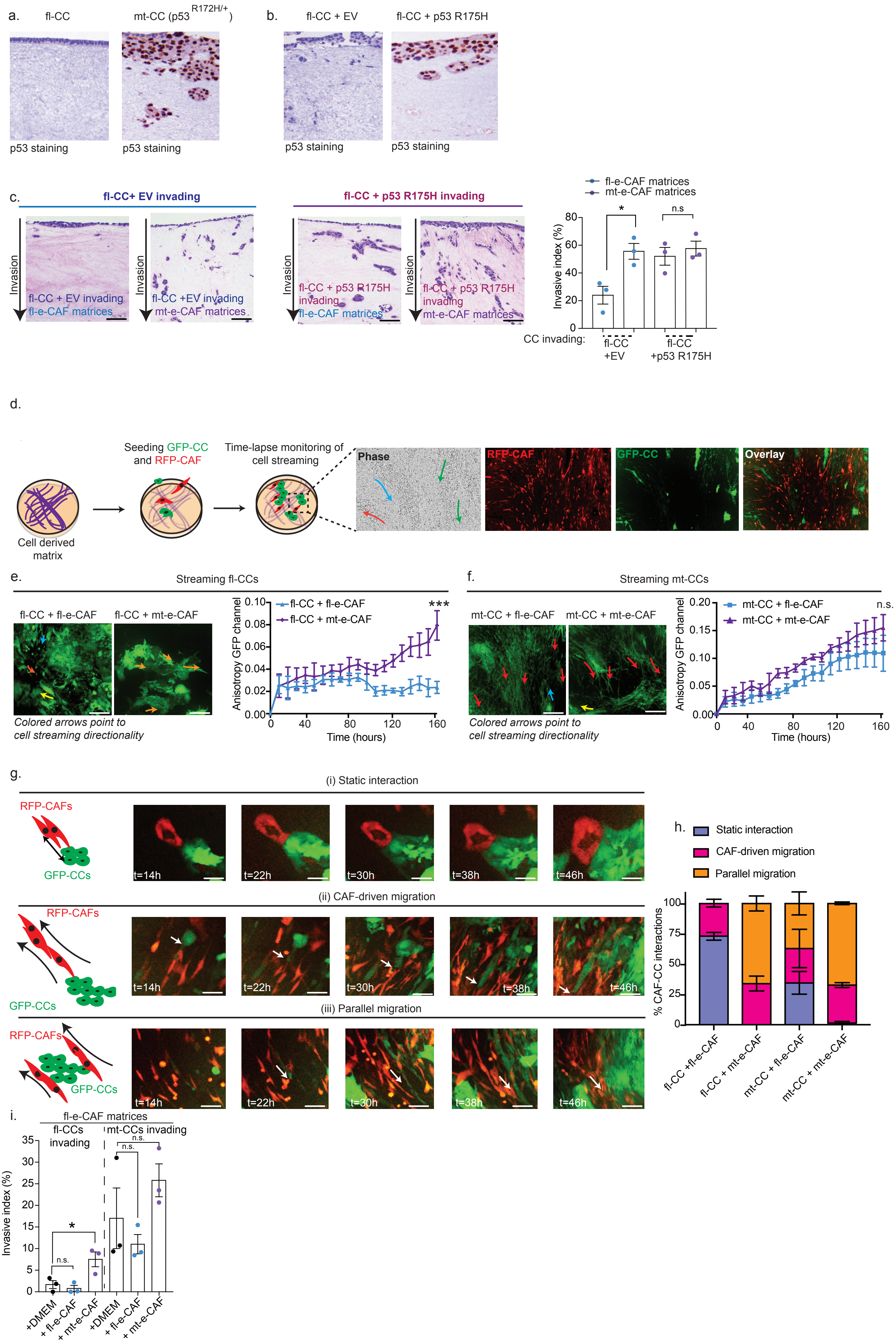

Supplementary figure 2. mt-e-CAFs create a pro-invasive environment.

a. Representative images of p53 staining (PAb421 antibody) of fl-CCs, mt-CCs (p53R172H/+) and of b. fl-CCs + empty vector (EV) and fl-CCs +p53R175H seeded on a collagen matrix. c. Representative H&E images and quantification of invasive index of fl-CCs + EV or fl-CCs + p53R175H invading into fl-e-CAFs (blue bars) or into mt-e-CAFs matrices (purple bars). Scale bar: 200 μm, n=3 biological repeats with three technical replicates per biological repeat. d. Schematic representation of streaming monitoring on cell-derived matrices and representative images of phase, RFP and GFP imaging of cancer cells and CAFs streaming onto a CDM. e. Representative GFP images and quantification of fl-CCs streaming over time and in the presence of fl-e-CAFs (blue line) or of mt-e-CAFs (purple line). Scale bar: 100 μm, n=3 biological repeats with three technical replicates per biological repeat. f. Representative GFP images and quantification of mt-CCs streaming over time and in the presence of fl-e-CAFs (blue line) or of mt-e-CAFs (purple line). Scale bar: 100 μm, n=3 biological repeats with three technical replicates per biological repeat. g. Schematic representation and representative images of (i) static interactions, (ii) CAF-driven migration and (iii) parallel migration occurring during cell streaming on CDMs. Scale bar: 5 μm. h. Quantification of the mode of streaming followed by fl-CCs or by mt-CCs in the presence of fl-e-CAFs or of mt-e-CAFs during streaming on CDMs. n=3 biological repeats with three technical replicates per biological repeat. i. Quantification of invasive index for cells seeded onto matrices generated by fl-e-CAFs and in the presence of DMEM control, fl-e-CAFs or mt-e-CAFs in the liquid phase. Relates to Fig. 2f, g. Individual data points are presented with mean values and SEM. * $p < 0.05$, *** $p<0.001$.

Supplementary figure 3

a.

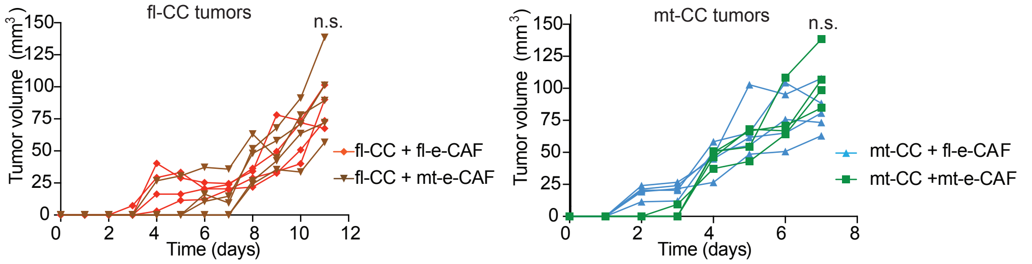

b.

Scoring system local invasion index

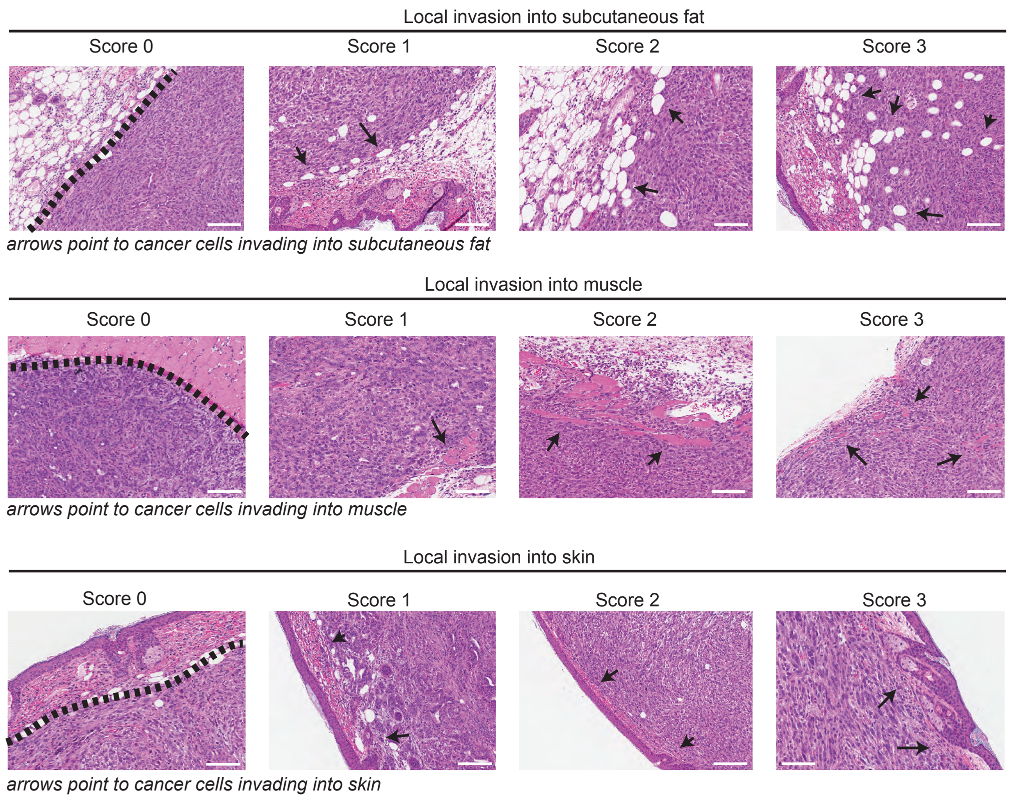

c.

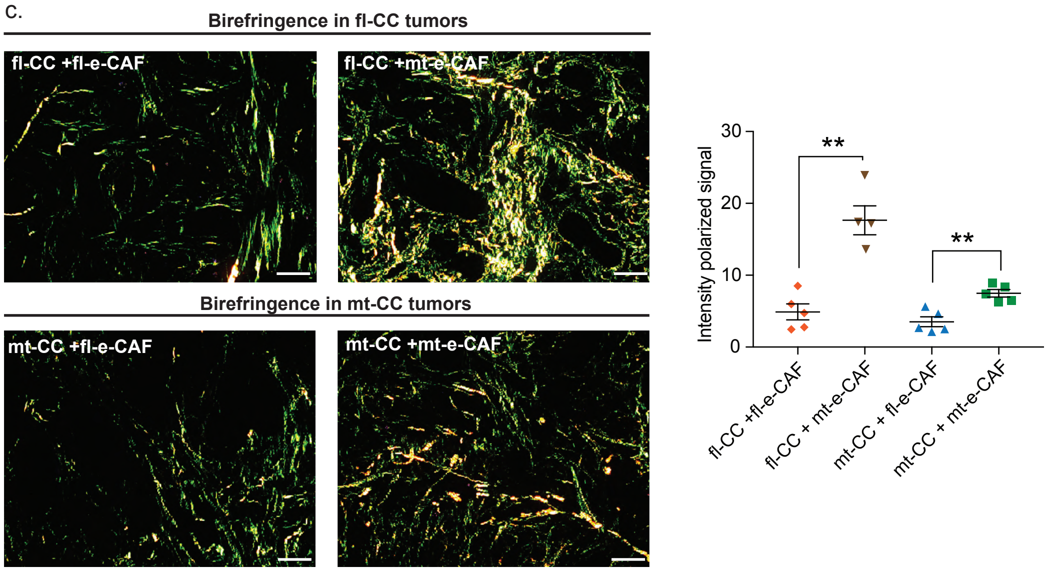

Supplementary figure 3. mt-e-CAFs drive local invasion and enhance collagen remodeling *in vivo*. a. Quantification of tumor volume in subcutaneous xenografts generated by fl-CCs injected with fl-e-CAFs (n=5 mice) or with mt-e-CAFs (n=4 mice), or by mt-CCs injected with fl-e-CAFs (n=5 mice) or with mt-e-CAFs (n=5 mice). b. Representative H&E images of subcutaneous xenografts depicting the scoring system employed to quantify local invasion into subcutaneous fat, muscle and skin. Relates to Figure 3. Scale bar: 300 µm. e-g. c. Representative images of polarized imaging of picrosirius red staining 3and quantification of collagen birefringence in subcutaneous xenografts. Scale bar: 50 µm, n=5 mice for mice injected fl-CCs+ fl-e-CAFs, with mt-CCs + fl-e-CAFs and with mt-CCs with mt-e-CAFs; n=4 mice for mice injected with fl-CCs + mt-e-CAFs. Individual data points are presented with mean values and SEM. ** $p < 0.01$.

Supplementary Figure 4

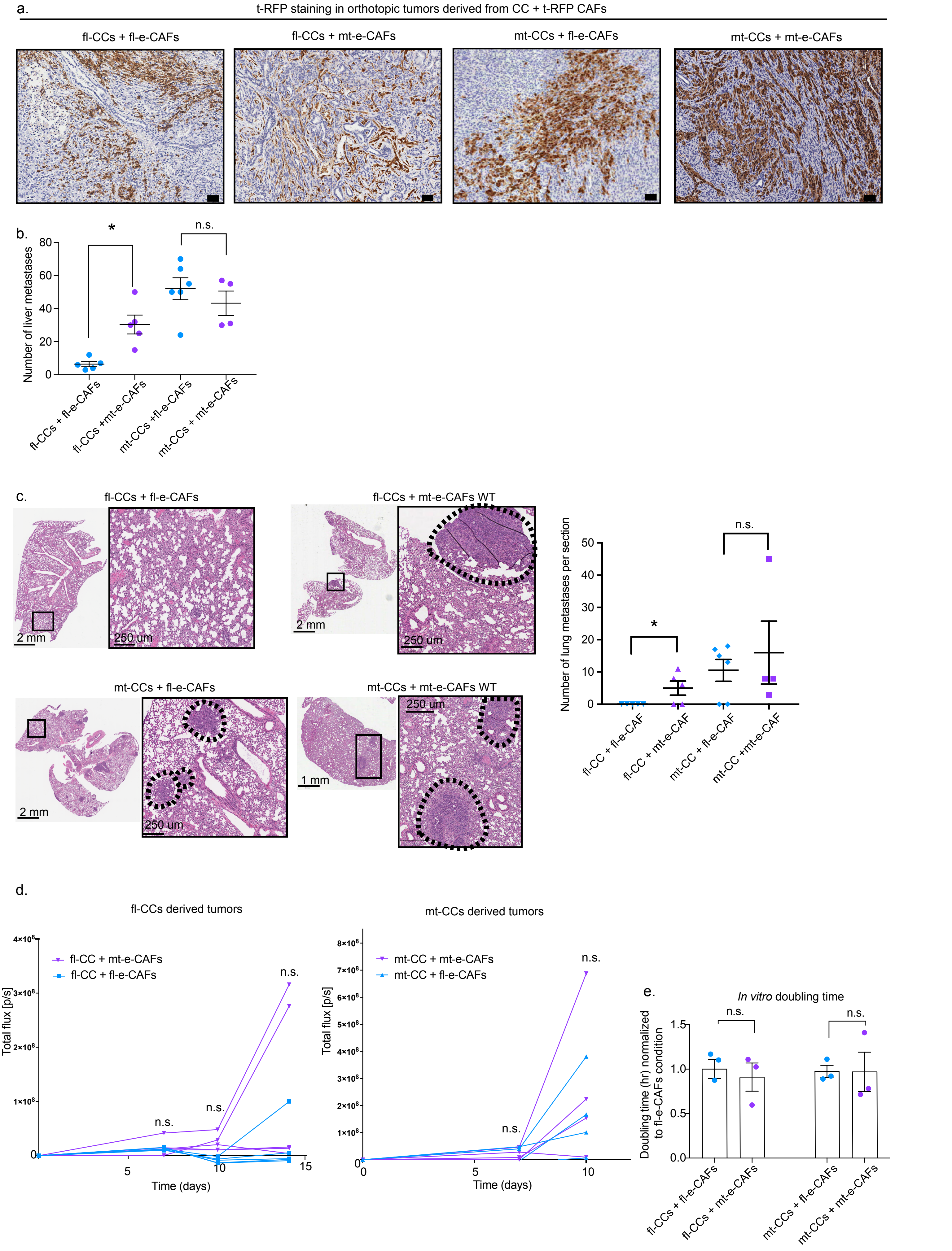

Supplementary figure 4: mt-e-CAFs increase metastatic burden in the orthotopic model.

a. Representative images of t-RFP staining to identify t-RFP-CAFs in pancreatic tumors derived from fl-CCs injected with fl-e-CAFs or with mt-e-CAFs, or derived from mt-CCs injected with fl-e-CAFs or with mt-e-CAFs. Scale bar: 200 µm. b. Quantification of the number of metastases visible on the liver at endpoint, c. representative images and quantification of lung metastases, d. quantification of luciferase signal in mice orthotopically injected with fl-CCs with fl-e-CAFs or with mt-e-CAFs, or orthotopically injected with mt-CCs injected with fl-e-CAFs or with mt-e-CAFs. e. *In vitro* doubling time of fl-CCs or mt-CCs co-cultured with either fl-e-CAFs or mt-e-CAFs. CAFs were treated with mitomycin c prior to co-culture with cancer cells. n=3 biological repeats with three technical replicates per biological repeat. Individual data points are presented with mean values and SEM. * $p < 0.05$.

Supplementary figure 5

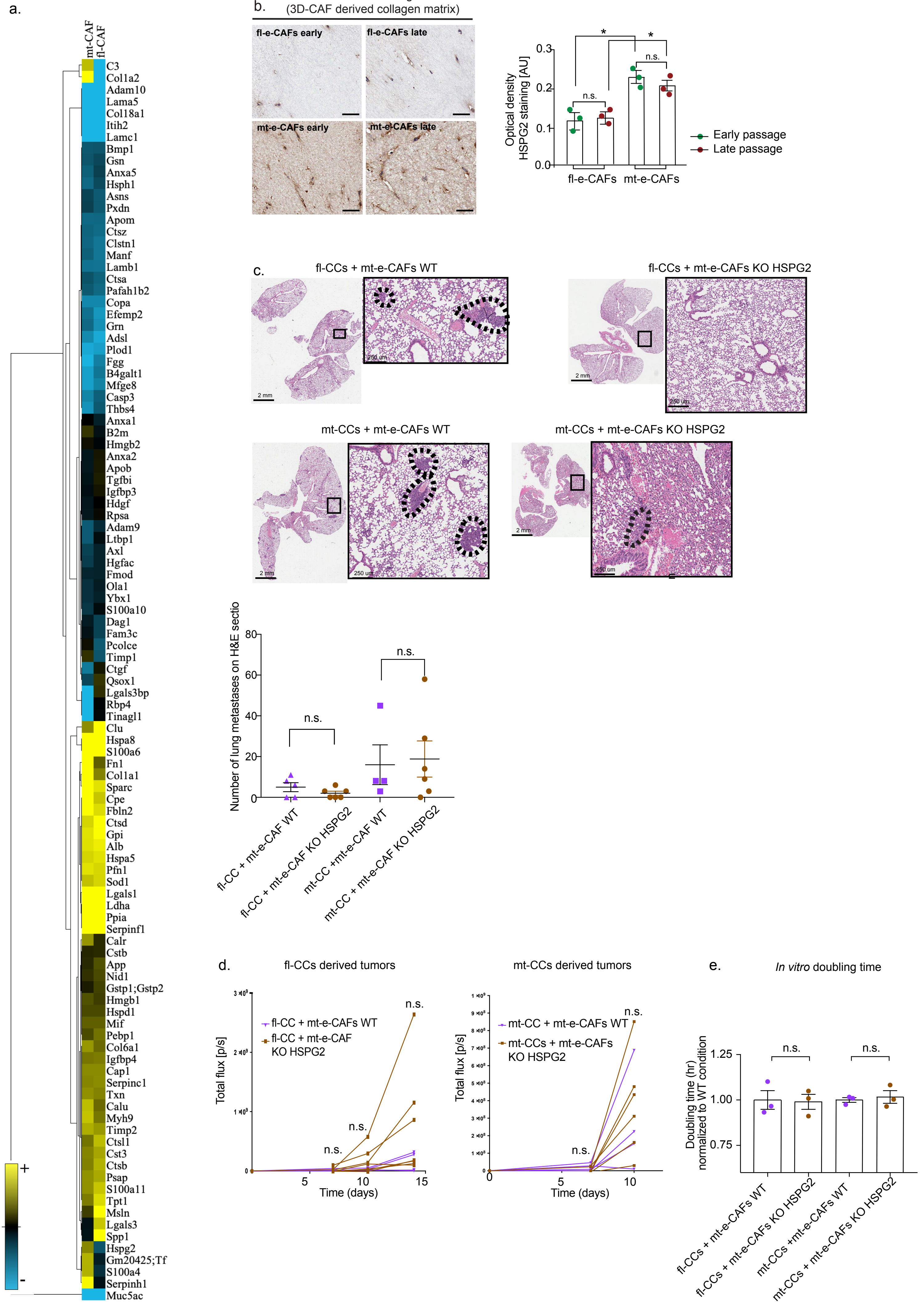

Supplementary figure 5: Orthotopic mt-CCs growth and progression upon loss of HSPG2 in mt-e-CAFs.

a. Heat map of proteomics analysis of the secretome of fl-e-CAFs and mt-e-CAFs (relates to Fig. 4a). b. Representative images and quantification of HSPG2 staining in matrices generated by fl-e-CAFs and mt-e-CAFs at early and late passages. c. Representative images and quantification of lung metastases, d. luciferase signal in mice orthotopically injected with fl-CCs with mt-e-CAFs WT or mt-e-CAFs KO HSPG2, or in mice orthotopically injected with mt-CCs with mt-e-CAFs WT or mt-e-CAFs KO HSPG2. e. *In vitro* doubling time of fl-CCs or mt-CCs co-cultured with either mt-e-CAFs WT or mt-e-CAFs KO HSPG2. CAFs were treated with mitomycin-c prior to co-culture with cancer cells. Data are presented as mean with SEM, n=3 biological repeats with three technical replicates per biological repeat. Individual data points are presented with mean values and SEM. * $p < 0.05$.

Supplementary figure 6

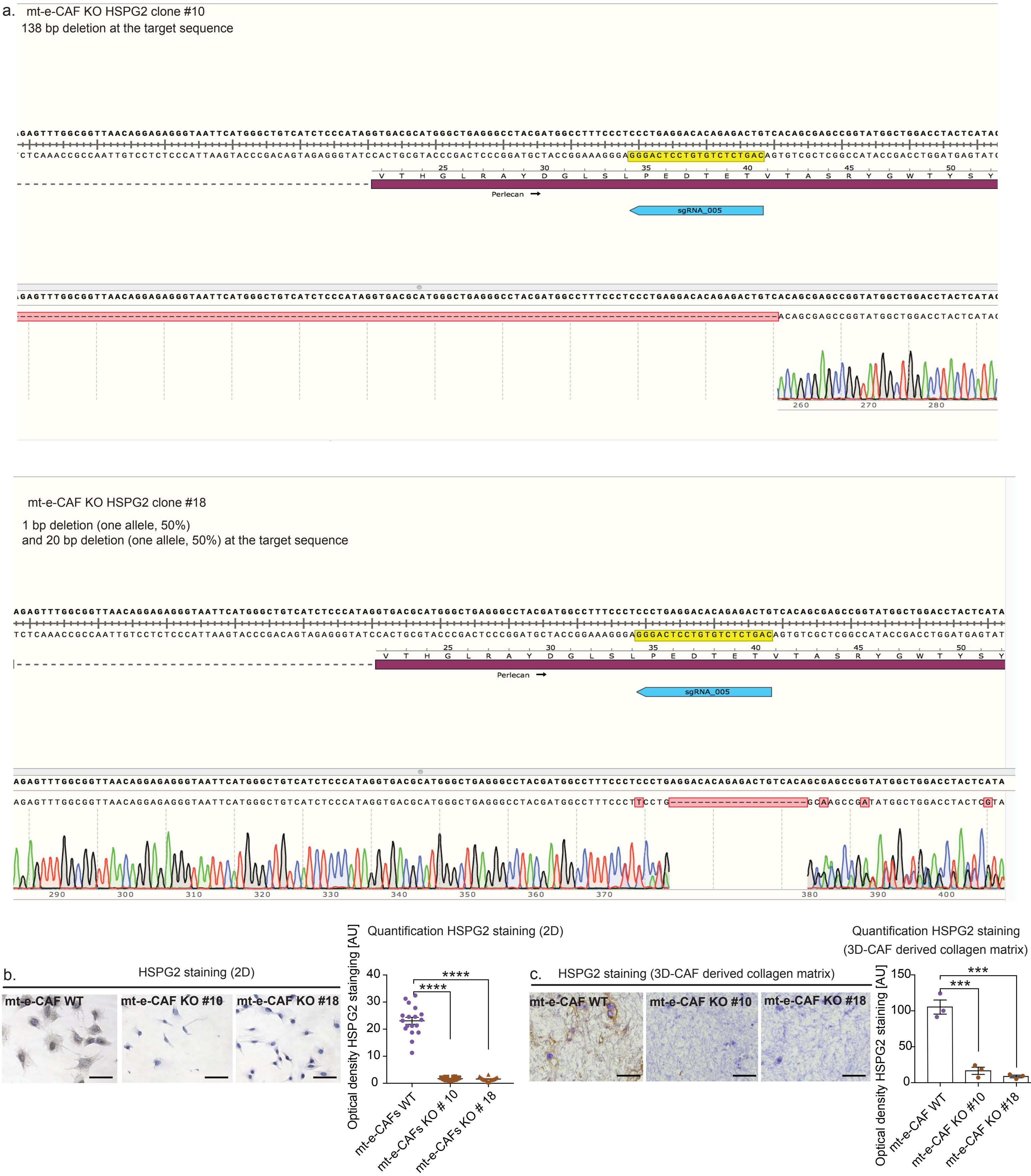

Supplementary figure 6. Depletion of HSPG2 in clones isolated from mt-e-CAFs KO HSPG2.

a. Sequencing of HSPG2 in clones 10 and 18 isolated from mt-e-CAFs KO HSPG2. b. HSPG2 staining and quantification in mt-e-CAFs WT or in clones 10 and 18 isolated from mt-e-CAF KO HSPG2 and cultured in 2D ($n>30$ cells analyzed per condition) or in c. collagen matrices ($n=3$ biological repeats with three technical replicates per repeat). Scale bars: 100 μm. Individual data points are presented with mean values and SEM, $n=3$ biological repeats with three technical replicates per repeat. *** $p<0.001$, **** $p<0.0001$.

Supplementary figure 7

a.

	GS follow link to MSigDB	SIZE	ES	NES	NOM p-val	FDR q-val
1	HALLMARK_EPITHELIAL_MESENCHYMAL_TRANSITION	192	0.58	2.19	0.000	0.000
2	HALLMARK_ANGIOGENESIS	35	0.60	1.73	0.004	0.011
3	HALLMARK_UV_RESPONSE_DN	138	0.46	1.69	0.002	0.013
4	HALLMARK_APOPTOSIS	154	0.42	1.57	0.003	0.038
5	HALLMARK_ALLOGRAFT_REJECTION	181	0.41	1.56	0.003	0.033
6	HALLMARK_INTERFERON_ALPHA_RESPONSE	83	0.46	1.55	0.011	0.031
7	HALLMARK_INFLAMMATORY_RESPONSE	189	0.40	1.52	0.000	0.036
8	HALLMARK_TNFA_SIGNALING_VIA_NFKB	191	0.39	1.49	0.002	0.042
9	HALLMARK_HYPOXIA	188	0.38	1.45	0.000	0.054

b.

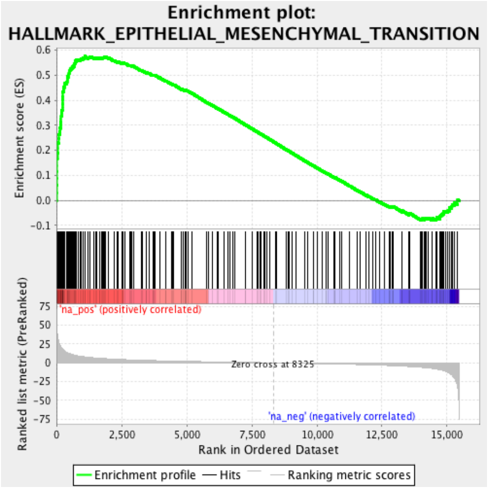

c.

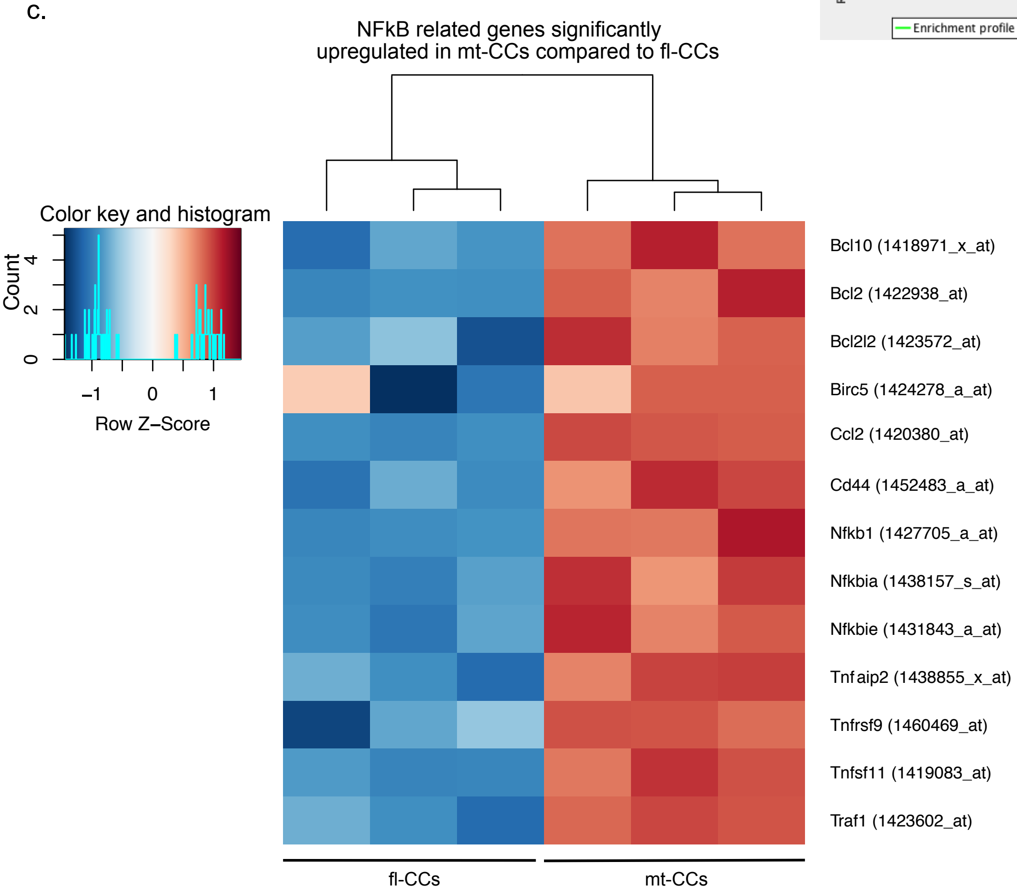

d.

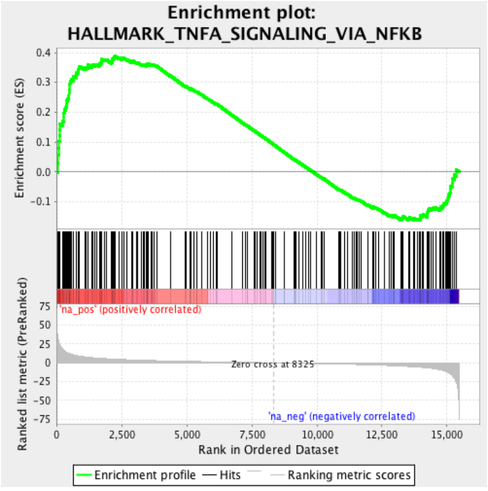

e. Analysis workflow in QuPath

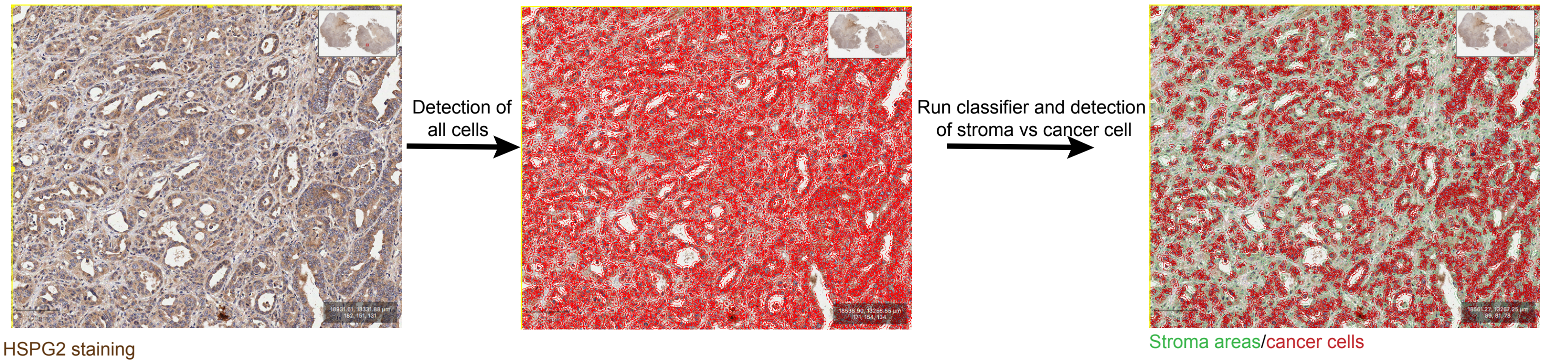

Supplementary figure 7: Paracrine signaling between mt-CCs and CAFs is partly mediated by NFκB.

a. Table of hallmarks enriched in mt-CCs compared to fl-CCs as identified via GSEA. b. Enrichment plot of hallmark of epithelial-to-mesenchymal (EMT) signature in mt-CCs compared to fl-CCs. c. Heatmap of microarray analysis of NFκB-target genes in fl-CCs versus mt-CCs. d. Enrichment plot of hallmark of TNFa signalling via NFκB in mt-CCs compared to fl-CCs. Also see Supplementary Table 2 and GEO accession number GSE123646. n=3 independent replicates per cell line. e. Workflow in QuPath for identification of cancer cells versus stromal areas in pancreatic tumors isolated from GEMMs and stained for HSPG2. Relates to Fig. 6g.

Supplementary figure 8

Staining and quantification after 24h of treatment

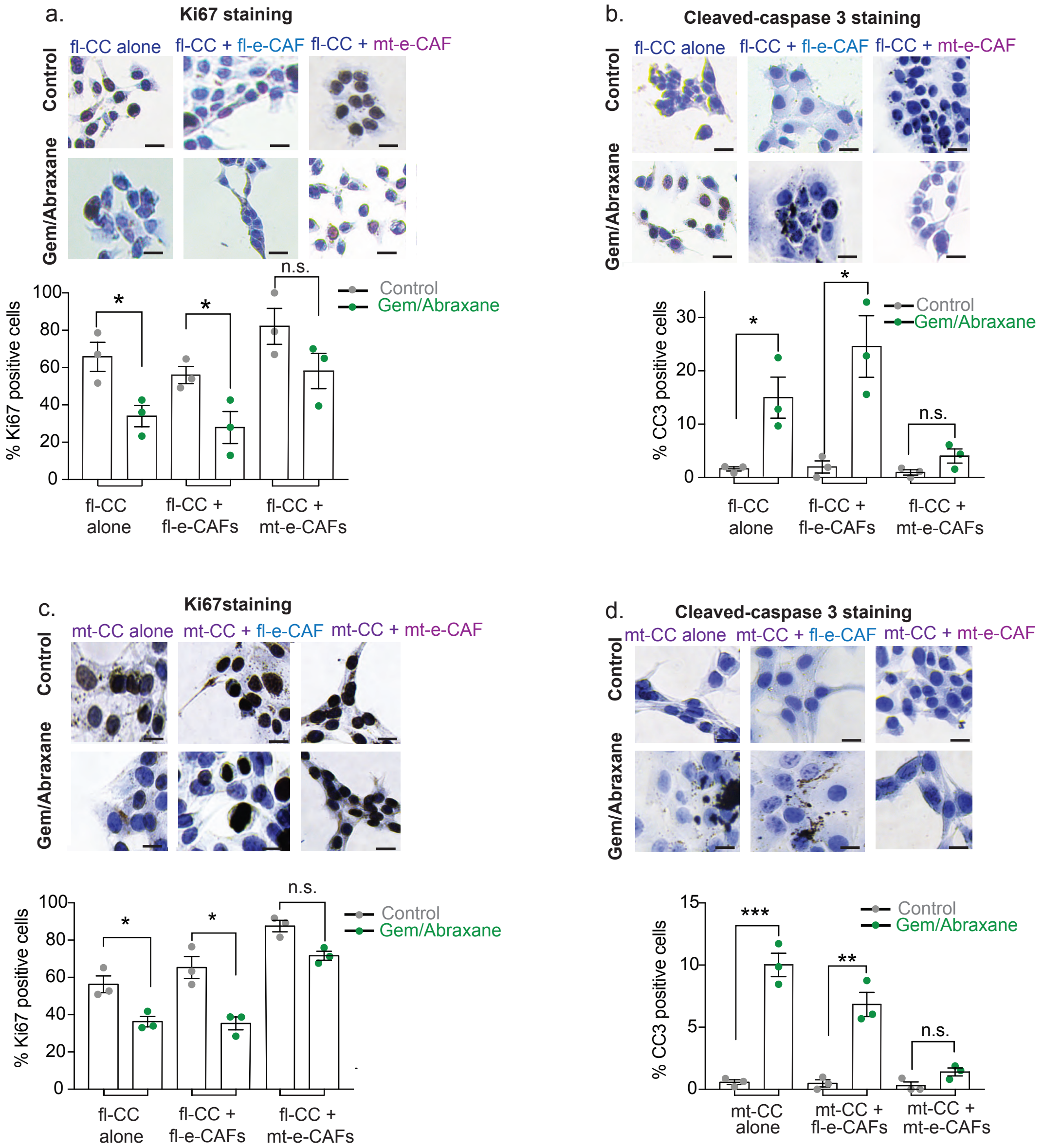

Supplementary figure 8. Immunocytochemistry analysis of cancer cell response to gemcitabine/Abraxane treatment.

a. Representative images and quantification of cleaved-caspase 3 (CC3) staining and b. Ki67 staining in fl-CCs cultured alone, co-cultured with fl-e-CAFs or with mt-e-CAFs and treated with control or with gemcitabine/Abraxane for 24h. c. Representative images and quantification of CC3 staining and d. Ki67 staining in mt-CCs cultured alone, co-cultured with fl-e-CAFs or with mt-e-CAFs and treated with control or with gemcitabine/Abraxane for 24h. Scale bar: 50 µm, n=3 biological repeats with 1 technical replicate per repeat. Individual data points are presented with mean values and SEM. * $p < 0.05$, ** $p < 0.01$, *** $p<0.001$.
