# Supplementary material for: CAF hierarchy driven by pancreatic cancer cell p53-status creates a pro-metastatic and chemoresistant environment via perlecan
Source: Nat Commun. 2019 Aug 12;10:3637. doi: 10.1038/s41467-019-10968-6 (PMC6691013; doi:10.1038/s41467-019-10968-6)
Supplement: Supplementary file 2 — Description of Additional Supplementary Files [file 41467_2019_10968_MOESM2_ESM.docx]

**Description of Supplementary Files**

**File Name:** Supplementary Data 1

**Description:** List of secreted proteins displaying a significant difference between fl-e-CAFs and mt-e-CAFs. n=5, data are given as log2 values. Relates to Fig. 4a.

**File Name:** Supplementary Data 2

**Description:** List of genes identified via microarray transcript expression profiling in fl-CCs compared to mt-CCs.

**File Name:** Supplementary Data 3

**Description:** List of primers utilized for RT-qPCR analysis of HSPG2 mRNA levels.

**File Name:** Supplementary Data 4

**Description:** Program utilized for RT-qPCR analyses.
