# Supplementary material for: CAF hierarchy driven by pancreatic cancer cell p53-status creates a pro-metastatic and chemoresistant environment via perlecan
Source: Nat Commun. 2019 Aug 12;10:3637. doi: 10.1038/s41467-019-10968-6 (PMC6691013; doi:10.1038/s41467-019-10968-6)
Supplement: Supplementary file 7 — Reporting summary [file 41467_2019_10968_MOESM7_ESM.pdf]

nature research

Corresponding author(s): Paul Timpson

Last updated by author(s): May 7, 2019

Reporting Summary

Nature Research wishes to improve the reproducibility of the work that we publish. This form provides structure for consistency and transparency in reporting. For further information on Nature Research policies, see Authors & Referees and the Editorial Policy Checklist.

Statistics

For all statistical analyses, confirm that the following items are present in the figure legend, table legend, main text, or Methods section.

n/a	Confirmed	
☐	☒	The exact sample size (*n*) for each experimental group/condition, given as a discrete number and unit of measurement
☐	☒	A statement on whether measurements were taken from distinct samples or whether the same sample was measured repeatedly
☐	☒	The statistical test(s) used AND whether they are one- or two-sided<br>*Only common tests should be described solely by name; describe more complex techniques in the Methods section.*
☐	☒	A description of all covariates tested
☐	☒	A description of any assumptions or corrections, such as tests of normality and adjustment for multiple comparisons
☐	☒	A full description of the statistical parameters including central tendency (e.g. means) or other basic estimates (e.g. regression coefficient) AND variation (e.g. standard deviation) or associated estimates of uncertainty (e.g. confidence intervals)
☒	☐	For null hypothesis testing, the test statistic (e.g. *F*, *t*, *r*) with confidence intervals, effect sizes, degrees of freedom and *P* value noted<br>*Give P values as exact values whenever suitable.*
☒	☐	For Bayesian analysis, information on the choice of priors and Markov chain Monte Carlo settings
☒	☐	For hierarchical and complex designs, identification of the appropriate level for tests and full reporting of outcomes
☒	☐	Estimates of effect sizes (e.g. Cohen's *d*, Pearson's *r*), indicating how they were calculated

Our web collection on statistics for biologists contains articles on many of the points above.

Software and code

Policy information about availability of computer code

Data collection	Luciferase signal in vivo was collected using Living Image Software (PerkinElmer), SHG, FLIM and immunofluorescence signals were collected using Leica Application Suite (LAS X), IHC staining were imaged using Leica Application Suite (LAS X) or on an Aperio Slide Scanner.
Data analysis	GLCM analysis was performed using previously published code (Vennin et al., Science Translational Medicine 2017), the code for FLIM analysis was developed by Dr. Aleš Benda and as previously published ((Vennin et al., Science Translational Medicine 2017). Quantification of staining and collagen birefringence was performed using Fiji (version 2.0.0-rc-68/1.52e), while SHG intensity was quantified using Matlab (MathWorks). For microarray analyses, differential expression between experimental groups was performed using Partek® Genomics Suite® software, Copyright ©; Partek Inc., St. Louis, MO, USA. Mouse tumors stained for perlecan were analysed using QuPath (0.1.2). For proteomic analyses, raw data were processed using MaxQuant version 1.5.8.3. Statistical analyses were performed using Prism GraphPad (version 7.0d).

For manuscripts utilizing custom algorithms or software that are central to the research but not yet described in published literature, software must be made available to editors/reviewers. We strongly encourage code deposition in a community repository (e.g. GitHub). See the Nature Research guidelines for submitting code & software for further information.

Data

Policy information about availability of data

All manuscripts must include a data availability statement. This statement should provide the following information, where applicable:

- Accession codes, unique identifiers, or web links for publicly available datasets
- A list of figures that have associated raw data
- A description of any restrictions on data availability

All relevant data supporting the findings of this study are available in the article and its supplementary information files and from the corresponding author upon reasonable request. Microarray data are freely available from GEO GSE123646. GLCM analysis was performed using previously published code (Vennin et al.,

Science Translational Medicine 2017), the code for FLIM analysis was developed by Dr. Aleš Benda and as previously published Vennin et al., Science Translational Medicine 2017).

Field-specific reporting

Please select the one below that is the best fit for your research. If you are not sure, read the appropriate sections before making your selection.

☒ Life sciences ☐ Behavioural & social sciences ☐ Ecological, evolutionary & environmental sciences

For a reference copy of the document with all sections, see nature.com/documents/nr-reporting-summary-flat.pdf

Life sciences study design

All studies must disclose on these points even when the disclosure is negative.

Sample size	For in vivo experiments, determination of the number of mice required to detect statistical significance was performed in line with 3Rs requirements. For changes in local invasion, we estimated an incidence of local invasion in ~75% of mice injected with mt-CCs (based on previously published work). Changes in the incidence of metastasis to ~25% was expected, and the predicted standard deviation was of 20%. Using these figures, and type-1 error alpha = 0.05, we could identify a difference in local invasion to a power of beta=80% at a significance level of 5% in cohorts of 4 mice per group.<br>For in vitro experiments, data presented in the manuscript represent three biological repeats with three technical replicates.
Data exclusions	For in vivo experiments, mice that did not develop tumors upon cancer cell transplantation, or that were sacrificed due to causes not related to tumor burden were excluded from the study.
Replication	All in vitro experiments were performed as three independent biological repeats with three technical replicates. Reproducibility of the results was tested using statistical analyses (see supplementary information of the manuscript).<br>All in vivo experiments were performed using at least 4 mice per arm, and reproducibility of the results was tested using statistical analyses (see supplementary information of the manuscript).
Randomization	Mice were randomized based on weight at the time of tumor cell transplantation.
Blinding	Researchers were blinded for quantification of local invasion in subcutaneous tumor.

Reporting for specific materials, systems and methods

We require information from authors about some types of materials, experimental systems and methods used in many studies. Here, indicate whether each material, system or method listed is relevant to your study. If you are not sure if a list item applies to your research, read the appropriate section before selecting a response.

Materials & experimental systems

n/a	Involved in the study	
☐	☒	Antibodies
☐	☒	Eukaryotic cell lines
☒	☐	Palaeontology
☐	☒	Animals and other organisms
☒	☐	Human research participants
☒	☐	Clinical data

Methods

n/a	Involved in the study	
☒	☐	ChIP-seq
☐	☒	Flow cytometry
☒	☐	MRI-based neuroimaging

Antibodies

Antibodies used	The following antibodies were used in the study: anti-EpCAM-PerCP/Cy5.5 (BioLegend®, Clone: G8.8), anti-CD140a-APC (BioLegend®, Clone: APA5) and anti-Podoplanin-PE (GP38) BioLegend®, Clone: 8.1.1), ACTA2 Abcam #ab5694; pMLC (Ser19) (Cell Signaling, #3671), pMYP1 (Thr696) (Millipore #ABS45), actin (Sigma Aldrich #A5441), Ki67 (ThermoScientific), cleaved-caspase 3 Cell Signalling #9661; Thermo Fischer, A7L6 HSPG2 (mAb targeting domain IV of mouse HSPG2, Merck Millipore; CCN1 (anti-HSPG2 antibody, developed by the Whitelock laboratory) and t-RFP (Evrogen #AB233), E-Cadherin (BD Biosciences #610182); FAP (Abcam #53066); keratin 19 (DSHB USA), Alexa488- or AlexaCy5-coupled secondary antibody (Jackon ImmunoResearch Laboratories Inc.), IκBα (Cell signalling #9246)and pIκBα (S32) (Cell Signaling #9246)
Validation	Validation of the primary antibodies were stated on the manufacturer's website. The CCN1 antibody produced by the Whitelock lab was validated in Shu et al., 2016.

Eukaryotic cell lines

Policy information about cell lines

Cell line source(s)	CAFs were isolated as described in the method section of our manuscript. Cancer cells were isolated from KPC and KPflC pancreatic tumors, as previously described (Morton, PNAS, 2010).
Authentication	The cancer cell lines were previously authenticated in Timpson et al., Cancer Research 2011
Mycoplasma contamination	All cell lines were tested negative for mycoplasma.
Commonly misidentified lines (See ICLAC register)	No commonly misidentified cell lines were used in the study.

Animals and other organisms

Policy information about studies involving animals; ARRIVE guidelines recommended for reporting animal research

Laboratory animals	Female BALB/c-Fox1nuAusb mice, between 6 weeks and 10 weeks old at the time of injections were used, as well as female NOD/SCID/ILR2γ mice, between 6 weeks and 10 weeks old at the time of injection.
Wild animals	The study did not contain wild animals.
Field-collected samples	The study did not contain field-collected samples.
Ethics oversight	The animal experiments were defined according to Garvan Ethics Committee guidelines (16/13 protocol).

Note that full information on the approval of the study protocol must also be provided in the manuscript.

Flow Cytometry

Plots

Confirm that:

- [ ] The axis labels state the marker and fluorochrome used (e.g. CD4-FITC).
- [ ] The axis scales are clearly visible. Include numbers along axes only for bottom left plot of group (a 'group' is an analysis of identical markers).
- [ ] All plots are contour plots with outliers or pseudocolor plots.
- [ ] A numerical value for number of cells or percentage (with statistics) is provided.

Methodology

Sample preparation	*Describe the sample preparation, detailing the biological source of the cells and any tissue processing steps used.*
Instrument	*Identify the instrument used for data collection, specifying make and model number.*
Software	*Describe the software used to collect and analyze the flow cytometry data. For custom code that has been deposited into a community repository, provide accession details.*
Cell population abundance	*Describe the abundance of the relevant cell populations within post-sort fractions, providing details on the purity of the samples and how it was determined.*
Gating strategy	*Describe the gating strategy used for all relevant experiments, specifying the preliminary FSC/SSC gates of the starting cell population, indicating where boundaries between "positive" and "negative" staining cell populations are defined.*

- [ ] Tick this box to confirm that a figure exemplifying the gating strategy is provided in the Supplementary Information.
